# A Synthetic Biology Workflow Reveals Variation in Processing and Solubility of Nitrogenase Proteins Targeted to Plant Mitochondria, and Differing Tolerance of Targeting Sequences in a Bacterial Nitrogenase Assay

**DOI:** 10.3389/fpls.2020.552160

**Published:** 2020-09-10

**Authors:** Shoko Okada, Christina M. Gregg, Robert Silas Allen, Amratha Menon, Dawar Hussain, Vanessa Gillespie, Ema Johnston, Keren Byrne, Michelle Lisa Colgrave, Craig C. Wood

**Affiliations:** ^1^ Land and Water, Commonwealth Scientific and Industrial Research Organisation, Acton, ACT, Australia; ^2^ Agriculture and Food, Commonwealth Scientific and Industrial Research Organisation, Acton, ACT, Australia; ^3^ Agriculture and Food, Commonwealth Scientific and Industrial Research Organisation, St. Lucia, QLD, Australia; ^4^ Australian Research Council Centre of Excellence for Innovations in Peptide and Protein Science, Queensland Biosciences Precinct, St. Lucia, QLD, Australia

**Keywords:** nitrogenase, plant mitochondria, mitochondrial targeting, mitochondrial processing peptidase, protein solubility, *Klebsiella*

## Abstract

While industrial nitrogen fertilizer is intrinsic to modern agriculture, it is expensive and environmentally harmful. One approach to reduce fertilizer usage is to engineer the bacterial nitrogenase enzyme complex within plant mitochondria, a location that may support enzyme function. Our current strategy involves fusing a mitochondrial targeting peptide (MTP) to nitrogenase (Nif) proteins, enabling their import to the mitochondrial matrix. However, the process of import modifies the N-terminus of each Nif protein and may impact nitrogenase assembly and function. Here we present our workflow assessing the mitochondrial processing, solubility and relative abundance of 16 *Klebsiella oxytoca* Nif proteins targeted to the mitochondrial matrix in *Nicotiana benthamiana* leaf. We found that processing and abundance of MTP::Nif proteins varied considerably, despite using the same constitutive promoter and MTP across all Nif proteins tested. Assessment of the solubility for all MTP::Nif proteins when targeted to plant mitochondria found NifF, M, N, S, U, W, X, Y, and Z were soluble, while NifB, E, H, J, K, Q, and V were mostly insoluble. The functional consequence of the N-terminal modifications required for mitochondrial targeting of Nif proteins was tested using a bacterial nitrogenase assay. With the exception of NifM, the Nif proteins generally tolerated the N-terminal extension. Proteomic analysis of Nif proteins expressed in bacteria found that the relative abundance of NifM with an N-terminal extension was increased ~50-fold, while that of the other Nif proteins was not influenced by the N-terminal extension. Based on the solubility, processing and functional assessments, our workflow identified that *K. oxytoca* NifF, N, S, U, W, Y, and Z successfully met these criteria. For the remaining Nif proteins, their limitations will need to be addressed before proceeding towards assembly of a complete set of plant-ready Nif proteins for reconstituting nitrogenase in plant mitochondria.

## Introduction

Industrial nitrogen fixation has had a major contribution towards the Green Revolution, and subsequent unprecedented population growth (Smil, 1999). However, the increase in global use of synthetic nitrogen fertilizer has resulted in environmental pollution, contributing to algal blooms, greenhouse gas accumulation and the acidification of soil and water sources (Vitousek et al., 1997; Glibert et al., 2014). There have been several efforts in the past 50 years to look for alternative, more sustainable means to deliver reduced nitrogen to crops, including the use of artificial symbiosis and commensal free-living bacteria (Santi et al., 2013; Curatti and Rubio, 2014; Oldroyd and Dixon, 2014). More recently, advances in synthetic biology have reignited the possibility of generating transgenic crops that can fix their own nitrogen *via* direct engineering of nitrogenase into plants.

Nitrogenase is the enzyme that catalyses biological nitrogen fixation, the process of converting atmospheric nitrogen to ammonia, and is found exclusively in bacteria and archaea. The molybdenum-dependent nitrogenase consists of two proteins that are highly oxygen-sensitive: The MoFe protein, a heterotetramer of NifD and NifK, and the Fe protein, a homodimer of NifH (Georgiadis et al., 1992; Kim and Rees, 1992). NifDK is the catalytic centre and contains the iron-molybdenum cofactor ([MoFe_7_S_9_C]:homocitrate, FeMo-co) (Spatzal et al., 2011; Lancaster et al., 2011) and the P-cluster ([Fe_8_S_7_]) (Peters et al., 1997). The Fe protein is the obligate electron donor to the MoFe protein and contains a [Fe_4_S_4_]-cluster. In addition to the structural proteins, numerous other nitrogenase (Nif) proteins are involved in the maturation of the enzyme and assembly of the metalloclusters. These include NifB, E, M, N, Q, S, U, V, W, X, Y, Z, and a ferredoxin (FdxN) (reviewed by Burén et al., 2020). Furthermore, some diazotrophs contain Nif-specific electron transport proteins, NifF, a flavodoxin, and NifJ, an associated oxidoreductase (Mus et al., 2019).

**Figure 1:**
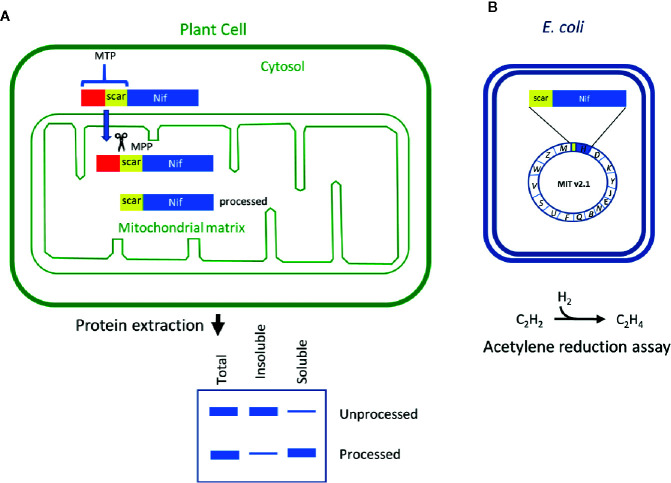
Schematic of the workflow to assess key features of Nif proteins targeted to plant mitochondria. **(A)** Translational fusions of the MTP to Nif proteins are expressed in *N. benthamiana* leaf to test preprotein processing and solubility. **(B)** Scar::Nif protein fusions are expressed in *E. coli* for function testing by acetylene reduction. MTP, “scar” peptide, Nif not to scale. MTP, mitochondrial targeting peptide; MPP, mitochondrial processing peptidase.

The mitochondrial matrix has been shown to be a suitable location to express some of the most oxygen-sensitive Nif components in a functional form, including the Fe protein and NifB (López-Torrejón et al., 2016; Burén et al., 2017a; Burén et al., 2019). Although direct expression of *nif* genes from the mitochondrial genome is an attractive approach, it is currently technically difficult to directly introduce transgenes into the mitochondrial genome and recover stable transgenic plants (Yoshizumi et al., 2018; MacMillan et al., 2019). In this study, we utilize the endogenous mitochondrial protein transport pathway for the expression of nuclear-encoded genes within the mitochondrial matrix (**Figure 1A**; reviewed by Ghifari et al., 2018). This process involves the use of mitochondrial targeting peptides (MTPs) as translational fusions at the N-terminus of each Nif protein (MTP::Nif). After translation in the cytosol the MTP::Nif protein is actively transported to the mitochondrial matrix through the outer and inner transmembrane import complexes. The MTP is cleaved within the mitochondrial matrix by the mitochondrial processing peptidase (MPP) and the remaining C-terminal peptide is folded into the mature protein. The MPP-dependent processing of the MTP results in residual amino acids at the N-terminus of the transgenic Nif proteins, and here we term this the ‘scar’ peptide. These N-terminal modifications could potentially impact the function of Nif proteins, and it is currently unknown if all Nif proteins can tolerate a scar peptide. The clearest examples of scar peptides being tolerated by the Nif proteins imported to yeast mitochondria are NifH, NifM, NifB, NifX, NifS, NifU, and FdxN (López-Torrejón et al., 2016; Burén et al., 2017a; Burén et al., 2019). All of these proteins are derived from *Azotobacter vinelandii*, with the exception of a NifB sourced from thermophilic methanogens to overcome solubility issues (Burén et al., 2017a; Burén et al., 2019). However, success in mitochondrial targeting of functional Nif components in yeast is yet to be replicated in plants.

Here we have assessed Nif proteins for their functional potential when targeted to plant mitochondria. Previously, 16 *Klebsiella oxytoca* Nif proteins were targeted to plant mitochondria by translationally fusing an MTP of 77 amino acids (AA), however neither the solubility of the targeted proteins or the impact of scar peptides on nitrogenase function were assessed at the time (Allen et al., 2017). In this study, the abundance, processing, and solubility of the 16 Nif proteins when targeted to plant mitochondria were evaluated. Furthermore, the functional impact of the N-terminal modifications to each Nif protein as a consequence of mitochondrial targeting was measured using a bacterial nitrogenase assay. Our workflow revealed that some *K. oxytoca* Nif proteins satisfied criteria for functional expression within plant mitochondria with the tested MTP, and others that will need further improvement in order to reconstitute nitrogenase in plant mitochondria.

## Results

### Design and Testing a 51 Amino Acid MTP for Targeting Nif Proteins to Plant Mitochondria

**Figure 2:**
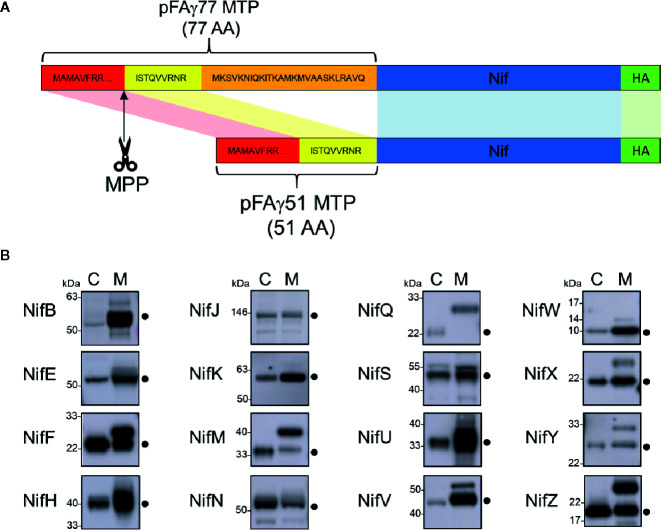
Design of the pFAγ51 MTP and assessment of its cleavage when fused to different Nif proteins expressed in plants. **(A)** Schematic of the truncation of the N-terminal amino acid sequence of the *A. thaliana* F1 ATPase γ subunit from 77 to 51 residues. The 26 amino acid residues in the orange section of pFAγ were removed. MTP, Nif, HA epitope tag are not to scale. MTP, mitochondrial targeting peptide; MPP, mitochondrial processing peptidase. **(B)** Western blot analysis (α-HA) of of the total protein extracts of individual pFAγ51::Nif::HA, pFAγ51::HA::NifK, 6×His::Nif::HA and 6×His::HA::NifK proteins transiently expressed in *N. benthamiana* leaf. Black dots point to the size of the correctly processed pFAγ51::Nif::HA protein. C, cytosolic expression; M, mitochondrial targeted. Panels of individual Nif proteins shown in B were extracted from full blot images presented in **Supplementary Figure 2**.

To target Nif proteins to the plant mitochondrial matrix, our previous work utilised a 77 AA peptide of the N-terminus of the ATP synthase γ subunit from *Arabidopsis thaliana* (pFAγ77) (Allen et al., 2017). Processing of pFAγ77 by the MPP resulted in a 35 AA residual ‘scar’ on the N-terminus of the NifH protein, the cleavage site of which was confirmed by proteomic analysis to be identical to that of the native ATP synthase γ subunit (Huang et al., 2009). However, introducing long N-terminal extensions to Nif proteins may impair function *via* steric hindrance. We therefore wanted to shorten the MTP to minimise the remaining scar yet retain targeting capability to the mitochondrial matrix. Previously Lee et al. (2012) showed that residues 52 to 77 of the original pFAγ77 were possibly not required for transporting and processing of green fluorescent protein to the mitochondrial matrix. Based on these observations we designed a shorter MTP with a length of 51 AA, here termed pFAγ51. pFAγ51 is predicted to leave a nine AA N-terminal extension after MPP processing that we term scar9 (amino acid sequence ISTQVVRNR, **Figure 2A**). To confirm the site of cleavage of pFAγ51 fused to Nif proteins we constructed a translational fusion of NifU with pFAγ51 at the N-terminus and a Twin-Strep-tag (Schmidt et al., 2013) at the C-terminus (pFAγ51::NifU::Twin-Strep-tag) using a modular Golden Gate assembly method (Weber et al., 2011). After agroinfiltration of pFAγ51::NifU::Twin-Strep-tag into *N. benthamiana* leaf, this protein was purified by affinity chromatography (**Supplementary Figure 1**) and subjected to proteomic analysis, which identified ISTQVVR as the dominant N-terminal peptide sequence. This result confirmed that the shortened MTP, pFAγ51, was functional and processed as predicted to leave a nine AA scar at the N-terminus of NifU.

### Most pFAγ51::Nif Proteins Are Targeted to and Processed Within the Plant Mitochondrial Matrix

We next wanted to assess if shortened MTP, pFAγ51, was able to target other *Klebsiella oxytoca* Nif proteins to the plant mitochondrial matrix. We generated expression constructs for 16 Nif proteins, each with translational fusions of pFAγ51 at the N-terminus and a HA epitope tag at the C-terminus resulting in the generic structure pFAγ51::Nif::HA (**Figure 2A**, plant expression constructs listed in **Supplementary Table 1**). NifK was the only protein for which a different construct was made, where the HA-tag was included at the N-terminus between pFAγ51 and NifK, since any C-terminal fusions render NifK non-functional (Yang et al., 2018). Expression of pFAγ51::NifD::HA has been reported as part of a separate study (Allen et al., 2020) and therefore was not included in this study. We constructed control expression plasmids to mimic the processed protein size for all Nif proteins by replacing pFAγ51 with 6×His. These 6×His::Nif::HA proteins are expected to be located to the cytosol. Both pFAγ51::Nif::HA and 6×His::Nif::HA were infiltrated, separately, in transient *N. benthamiana* leaf assays and the migration speeds of the expressed proteins were assessed by western blot analysis.

Comparison of each Nif protein targeted to either the mitochondrial matrix or the cytosol demonstrated that 15 of 16 Nif proteins targeted to the mitochondria using pFAγ51 were processed by MPP, albeit with variable efficiencies (**Figure 2B**, full blot images provided in **Supplementary Figure 2**). We observed three general classes of processing, either efficient, partial or no apparent cleavage. Efficient cleavage was found for eight pFAγ51::Nif::HA proteins (NifB, E, H, J, N, U, V, W) and pFAγ51::HA::NifK. Six pFAγ51::Nif::HA proteins (NifF, M, S, X, Y, Z) were partially cleaved, as evidenced by the presence of two HA-dependent signals, the faster band migrating at the speed of the corresponding 6×His::Nif::HA control and another slower band running at a speed consistent with the size of the unprocessed pFAγ51::Nif::HA (predicted unprocessed protein sizes provided in **Supplementary Table 1**). The only protein displaying no evidence of processing was pFAγ51::NifQ::HA, with only a signal found for a protein consistent with the unprocessed size (**Figure 2B**). For some pFAγ51::Nif::HA proteins, e.g., NifB, E, H, S, U, and Z there were additional signals at a higher molecular weight, which could arise from dimerization or oligomerization (**Supplementary Figures 2–4**), similar to what has been previously observed (Allen et al., 2017). In some instances, e.g., NifJ, we also observed degradation products (**Supplementary Figure 2**).

As each Nif protein had a HA epitope tag, it was possible to assess the relative abundance of each Nif protein expressed in *N. benthamiana* leaf. In general, we found that most of the pFAγ51::Nif::HA and pFAγ51::HA::NifK proteins were readily detectable, although their abundance varied (**Figure 2B**). For instance, pFAγ51::NifY::HA had a relatively low signal intensity whereas NifH, F, and U had the highest signal intensities.

### Assessing the Solubility of Nif Proteins Targeted to Plant Mitochondria

**Figure 3:**
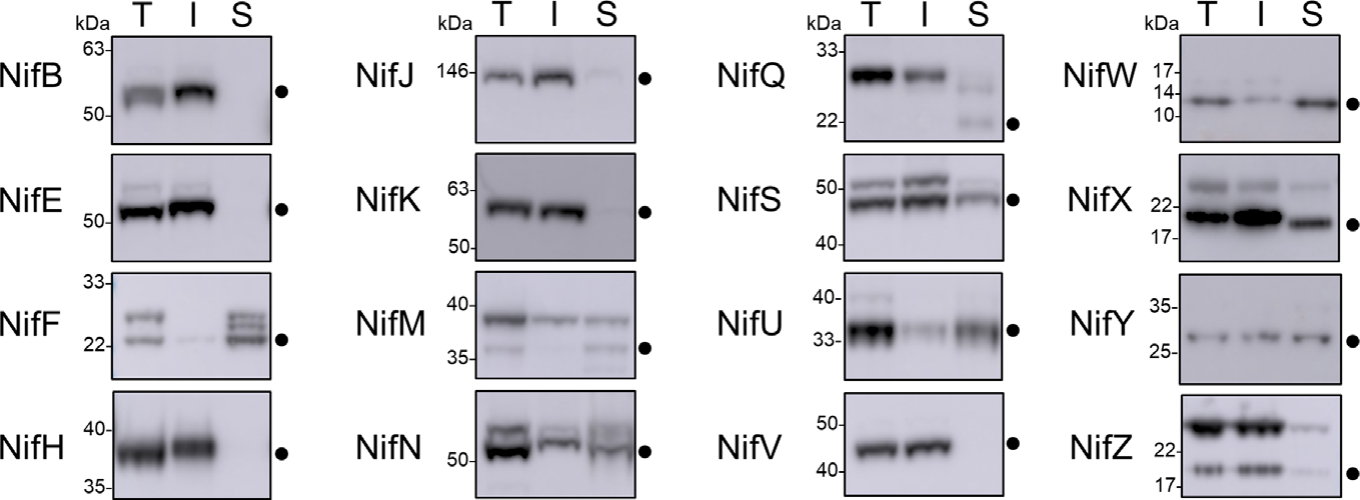
Assessment of the solubility of Nif proteins targeted to the mitochondria and cytosol of plants by western blot analysis (α-HA) of individual pFAγ51::Nif::HA and pFAγ51::HA::NifK proteins transiently expressed in *N. benthamiana* leaf. Black dots point to the size of the processed pFAγ51::Nif::HA and pFAγ51::HA::NifK protein. T, total protein; I, insoluble fraction; S, soluble fraction. Panels of individual Nif proteins shown were extracted from full blot images presented in **Supplementary Figure 3**.

As all Nif proteins need to be soluble for function we tested the solubility of the Nif proteins when targeted to the plant mitochondrial matrix. Total protein extracts of *N. benthamiana* leaf tissue individually expressing 15 pFAγ51::Nif::HA and pFAγ51::HA::NifK were separated into soluble and insoluble fractions and analyzed by western blot (**Figures 1A** and **3**, full blot image provided in **Supplementary Figure 3**). We found that the relative abundance of correctly processed Nif proteins in the soluble fraction varied. For example, NifF, M, N, S, and W were predominantly in the soluble fraction in both the correctly processed and unprocessed form. Conversely NifB, E, H, J, and V were not found in the soluble fraction, despite being correctly processed. We found a third band for NifF between the processed and unprocessed form, which was possibly an artefact of soluble protein extraction, as it was not detected in the western blot image of total protein. pFAγ51::NifQ::HA produced a faint band approximately the size of the correctly processed form in the soluble fraction, although it was not detectable in the western blot of total protein. This signal of pFAγ51::NifQ::HA in the soluble fraction may be due to an artefact of soluble protein extraction as seen with NifF. To assess if atmospheric oxygen affected Nif protein solubility the same 15 pFAγ51::Nif::HA and pFAγ51::HA::NifK proteins were isolated from infiltrated plants under anaerobic conditions and subjected to western blot analysis. We found that extraction of Nif proteins under anaerobic conditions did not change their solubility beyond the biological variation that we expected to see between different agroinfiltration sets (**Supplementary Figure 4**).

Since it has been reported that several *Klebsiella* Nif proteins have physical interactions that are suggested to provide mutual stability (Roberts et al., 1978), we coexpressed two combinations of Nif proteins that are known to physically interact – NifH and NifM, and NifE and NifN – which were targeted to the mitochondria of *N. benthamiana* leaf. We were particularly interested to see if NifE and NifH, both of which were insoluble in their processed form when targeted to the plant mitochondria individually (**Figure 3**) would become soluble when coexpressed with their respective physical partners. Coexpression of either Nif combination did not improve the solubility of any of the Nif protein counterparts (**Supplementary Figure 5**). Results on coexpression of NifD and NifK targeted to *N. benthamiana* leaf mitochondria have been reported in a separate publication (Allen et al., 2020).

### Testing Function of Nif Proteins Modified With an N-Terminal Extension

The import of Nif proteins to the mitochondrial matrix *via* MTP::Nif translational fusions results in short additions to the N-terminus of the Nif proteins. To assess the potential impact of these modifications on function, we used a bacterial-based assay. We used *Escherichia coli* because it is known to fix nitrogen when provided with 16 K*. oxytoca* *nif* genes in the form of a plasmid (Temme et al., 2012; Smanski et al., 2014; Yang et al., 2018), as we currently do not have a method to test nitrogenase function as a whole in a eukaryotic setting. The pFAγ51 MTP used in this study generates a nine AA N-terminal extension after cleavage by MPP in the mitochondrial matrix, as shown by the processing of pFAγ51::NifU::Twin-Strep-tag in *N. benthamiana*. We adopted the MIT v2.1 plasmid system in *E. coli* (Smanski et al., 2014) and fused the nine AA scar9 sequence, MSTQVVRNR, to the N-terminus of each Nif protein, mimicking the length and sequence of pFAγ51 after MPP cleavage. An example of the process is outlined with *scar9::nifH* replacing *nifH* within MIT v2.1 (**Figure 1B**). Each scar9::Nif protein was tested individually in separate MIT v2.1 plasmids in the same manner (bacterial expression constructs listed in **Supplementary Table 2**). It is worth noting that MIT v2.1 does not have *nifX* and therefore we did not test the impact of scar9 on this protein with respect to nitrogenase function. As a negative control we removed *nifH*, *D*, *K*, *Y*, *E*, *N*, and *J* from MIT v2.1 and made a non-functional plasmid, here termed ‘pB-ori’. As further controls we made other modifications, such as adding a HA epitope tag to the C-terminus of *nifK*, namely *NifK::HA*, or removing *nifM* from MIT v2.1 (cf. Howard et al., 1986; Lei et al., 1999), both of which resulted in the expected loss of nitrogenase function (**Table 1**). Function testing of the individual scar9::Nif proteins in *E. coli* by acetylene reduction showed that nitrogenase activity was retained with all 16 scar9::Nif proteins although there was variation in activity levels. Notably when activity was tested with scar9::NifJ it had over 250% of the positive control, and those with scar9::NifH, B and F were mildly increased (124%–109% activity relative to MIT v2.1). In contrast, the activities with scar9::NifE, N, S, Y, and Z were less than half compared to that of the positive control. The most negatively impacted function with the nine AA extension was scar9::NifM, which only retained approximately 10% activity of the positive control.

**Table 1:** Summary of mitochondrial processing efficiency and solubility of the processed form of pFAγ51::Nif proteins expressed in *Nicotiana benthamiana*, and effect of pFAγ51 nine amino acid “scar” (scar9) peptide translationally fused to individual Nif proteins on nitrogenase function tested in *Escherichia coli*.

N. benthamiana construct	Mitochondrial processing	Solubility of processed form	E. coli construct	E. coli function testing		
				% Activity of MIT v2.1	S.D.	n
n/a	n/a	n/a	MIT v2.1	100	33	8
pFAγ51::J	++	–	scar9::J	257	72	4
pFAγ51::H	++	–	scar9::H	124	12	4
pFAγ51::B	++	–	scar9::B	120	28	4
pFAγ51::D	n/a^#^	n/a^#^	scar9::D	110	9	2
pFAγ51::F	+	++	scar9::F	109	6	4
pFAγ51::Q	–	?*	scar9::Q	102	49	4
pFAγ51::W	++	+	scar9::W	57	26	4
pFAγ51::U	++	+	scar9::U	55	14	4
pFAγ51::V	++	–	scar9::V	52	18	4
pFAγ51::K	++	–	scar9::K	50	5	4
pFAγ51::Y	+	+	scar9::Y	44	12	4
pFAγ51::Z	+	+	scar9::Z	43	23	4
pFAγ51::S	+	+	scar9::S	39	5	4
pFAγ51::N	++	+	scar9::N	38	7	3
pFAγ51::E	++	–	scar9::E	33	2	4
pFAγ51::M	+	++	scar9::M	8	2	4
pFAγ51::X	+	+	scar9::X	n/a	n/a	n/a
n/a	n/a	n/a	*ΔnifM*	5	3	3
n/a	n/a	n/a	NifK::HA	0	1	3
n/a	n/a	n/a	p-ori (*ΔnifJDKYENJ*)	0	0	8

Values are presented as % acetylene reduction activity compared to MIT v2.1. pB-ori, negative control containing nifBQFUSVWZM; ▵nifM, NifM coding sequence removed from MIT v2.1; NifK::HA, HA epitope tag fused to the C-terminus of NifK; S.D., standard deviation (n=2-8). ++, efficiently processed or mostly soluble; + partially processed or partially soluble; - not processed or insoluble. ^#^Reported in Allen et al., 2020. *Solubility of pFAγ51::NifQ in N. benthamiana may be an artifact during soluble protein extraction.

To assess if the scar9 peptide had any influence on the relative abundance of each Nif protein in *E. coli*, we measured the relative protein abundance of the Nif proteins in *E. coli* containing the native and modified forms of MIT v2.1 using targeted proteomics. We also measured the relative abundance of a peptide specific to chloramphenicol acyltransferase (CAT), the coding gene of which was present in all MIT v2.1 plasmids. We found that the signal for the CAT peptide was relatively consistent (**Supplementary Figure 6**, **Supplementary Table 3**), indicating that the relative expression levels of this protein was similar across the different samples. As expected, we also found that peptide signals specific to NifM, peptides M-1 and -2, were not found in *E. coli* containing MIT v2.1 in which NifM was deleted. The most unexpected change was found for NifM, where the relative protein abundance was approximately ~50-fold higher in *E. coli* expressing scar9::NifM, relative to those expressing other scar9::Nif proteins (**Supplementary Figure 6**, **Supplementary Table 3**).

## Discussion

In this study, we assessed key prerequisites for targeting Nif proteins to plant mitochondria using a systematic approach. The properties tested were cleavage of the MTP by the MPP, and solubility and relative protein abundance of the Nif proteins in the plant mitochondrial matrix. We also investigate for the first time the tolerance of N-terminal scar sequences for nitrogenase function in *E. coli*. Collectively these assessments represent a testing workflow that can help identify potentially suitable MTP::Nif proteins, and at the same time expose problems with certain MTP::Nif protein combinations for plant mitochondrial expression. These plant- and bacterial-based assays identified seven Nif proteins, namely pFAγ51::NifF, N, S, U, W, Y, and Z, that we consider potentially sufficient for metabolic engineering of nitrogenase into plant mitochondria. Importantly, we found other MTP::Nif proteins to be either poorly processed, insoluble in plant mitochondria, or impaired in functional assays. The identification of these problematic MTP::Nif proteins will guide targeted improvements in the future.

The relative protein abundance, processing efficiency, and solubility of the 16 different MTP::Nif proteins varied, despite use of the same MTP and promoter for each plant expression construct. This variation illustrates how intrinsic properties of each Nif protein influence these attributes in plant mitochondria. Assuming that the expression levels of Nif proteins in plant mitochondria will need to reflect those in diazotrophic bacteria, future studies will need to adjust promoter strength and/or translation rates accordingly. For example, NifY was the least abundant in our experiments, and efforts are needed to improve expression levels to better mimic those found in naturally occurring systems (Poza-Carrión et al., 2014; Smanski et al., 2014).

A small proportion of MTP::Nif proteins were poorly cleaved by the MPP within the plant mitochondria, in particular MTP::NifQ. A potential reason for this may be that the preprotein is unable to enter the mitochondrial matrix due to the MTP::Nif protein being resistant to unfolding (Voos et al., 1993; Voos et al., 1996). We also found that most Nif proteins that were successfully cleaved within the mitochondrial matrix tended to accumulate to higher levels relative to their cytosolic counterparts, suggesting that mitochondrial processing may stabilize Nif proteins relative to those located in the cytosol.

Several MTP::Nif proteins were insoluble in the plant mitochondrial matrix, and there are numerous reasons that could underlie this, including association with membranes, incorrect folding, and interactions with other proteins. Notably the key protein NifH from *K. oxytoca* was among this set of insoluble proteins. Interestingly, *A. vinelandii* NifH and NifM when targeted to yeast mitochondria produced a functional Fe protein (López-Torrejón et al., 2016), showing that both these proteins were sufficiently soluble in yeast. In agreement with prior results, we found that *K. oxytoca* NifB was insoluble when targeted to plant mitochondria, as was described for *A. vinelandii* NifB when targeted to yeast and plant mitochondria (Burén et al., 2017a).

Overcoming these solubility issues, as well as suboptimal processing and abundance, will likely require a range of approaches. In yeast mitochondria, there is evidence that the import of transgenic cargoes can be improved by the use of longer MTPs (Wilcox et al., 2005). Therefore, screening MTPs of varying lengths may reveal certain MTP::Nif combinations that overcome problems with recalcitrant import. Using other bacterial or archaeal variants of the nitrogen fixation pathway may also improve mitochondrial targeting and MPP processing, solubility and ultimately activity of nitrogenase within mitochondria, as was shown for NifB (Burén et al., 2017a; Burén et al., 2019). Although this report concentrates on attributes of Nif proteins expressed individually, there may be combinations of Nif proteins that when expressed together improve protein abundance or solubility. Functional *A. vinelandii* Nif proteins have been successfully coexpressed within yeast mitochondria (López-Torrejón et al., 2016; Burén et al., 2017b; Burén et al., 2019), and having these protein combinations present in the one organelle may overcome problems that we report here. Finally, physically linking Nif proteins into larger multi-domain polyproteins (Yang et al., 2018; Allen et al., 2020) could assist in protein assembly and may overcome problems associated with relative abundance and solubility.

The function of nitrogenase may be impacted by scar residues originating from the MPP cleavage of the MTP. Yang et al. (2018) demonstrated that the addition of the tobacco etch virus (TEV) protease cleavage site to the C-terminus of NifK abolished activity, a result that agrees with the close interaction of the C-terminal region of NifK with NifD (Kim and Rees, 1992; Spatzal et al., 2011). Here we tested a nine AA extension on the N-terminus of 16 Nif proteins and found both positive and negative impacts on overall nitrogenase activity. Importantly we found that the key protein NifH supported nitrogen fixation with the N-terminal extension. NifH has three different functions, firstly, it donates electrons to NifDK, secondly, it is involved in the maturation of the P-clusters within NifDK, and thirdly it is involved in FeMo cofactor synthesis (reviewed in Hu and Ribbe, 2013). In a previous study, NifH isolated from yeast mitochondria was capable of donating electrons to NifDK (López-Torrejón et al., 2016). Our study demonstrates that the additional functions of NifH can also occur despite the addition of scar9 at its N-terminus. Examples of Nif proteins that had reduced activity with the N-terminal nine AA addition were NifE, N, and M. In the case of NifEN these proteins form a stable heterotetramer, but also interact with numerous other Nif proteins during the biogenesis of FeMo-co, including NifB, NifY, and NifH (Ribbe et al., 2014). The N-terminal extension on NifE and NifN that was tested in our study may have reduced nitrogenase function *via* steric hinderance within protein-protein interactions associated with NifEN. The most severe impact on nitrogenase function was found for scar9::NifM (~10% of control), but in that instance proteomic analysis found that the abundance of scar9::NifM was highly upregulated compared to other modified MIT v2.1 plasmids. Nitrogenase activity is highly sensitive to changes in Nif protein levels (reviewed in Martinez-Argudo et al., 2004) and for optimum nitrogenase activity, the stoichiometry of the numerous Nif proteins and their temporal expression needs to be tightly regulated (Poza-Carrión et al., 2014). Therefore, this misregulation of scar9::NifM, which may be specific to *E. coli* expression, may account for the decrease in nitrogenase activity rather than reflecting steric interference. Another consideration when testing the function with modified Nif proteins is their potential redundancy due to the presence of homologous proteins in the native host (*E. coli* in our case) that could substitute for function of the modified Nif protein. The *E. coli* K12 genome contains homologous genes for *nifFJSUV* (**Supplementary Table 4**). The *nifV* homologue, *leuA*, codes for 2-isopropylmalate synthase. Functional characterization of this enzyme (Hunter and Parker, 2014) suggests that it is unlikely to bind the NifV substrate α-ketoglutarate, and therefore not function as a homocitrate synthase. Harris et al. (1990) surveyed the redundancy of 14 *Klebsiella* *nif* gene products (*nifHDKTYENXUSVWMZ*) for apoMoFe protein production in *E. coli* and found *nifHDKS* to be essential, while *nifMYUWZ* were needed for maximum production. For of *nifJ* and *nifF*, Yang et al. (2014) found the *E. coli* orthologues of *nifJ* and *nifF* (*ydbK and fldA*, respectively) to partially substitute for nitrogenase activity, which suggests that they cannot fully replace the activities of the *Klebsiella* NifJ and NifF proteins in order to achieve optimal function. Therefore, for the non-essential Nif proteins that had reduced nitrogenase activity with the N-terminal modification (Nif U, W, Y, Z) we cannot determine the absolute effect of the added scar9 peptide to these proteins as the level of nitrogenase activity without those non-essential *nif* genes has not been determined in *E. coli*.

Although mitochondria are considered potentially suitable to support nitrogenase activity, impediments remain to successfully translocate all Nif proteins in a functional form to the organelle. This is not surprising considering the large span of evolutionary time separating the emergence of nitrogenase in bacteria from the origins of mitochondria in eukaryotes (Muller et al., 2012; Poole and Gribaldo, 2014). Our analysis uncovered some Nif proteins that we consider compatible with translocation to plant mitochondria with the MTP tested in this study and other Nif proteins that require further improvement. The experimental workflow outlined here can be applied to improve expression and targeting of all Nif proteins required with the eventual goal of assembling the entire pathway within plant mitochondria.

## Materials and Methods

### Construction of Plasmids for *Nicotiana benthamiana* Leaf Transient Expression

Plasmids for transient expression in *N. benthamiana* leaf were constructed using a modular cloning system with Golden Gate assembly (Weber et al., 2011). DNA parts as individual plasmids (Thermo Fisher Scientific, ENSA), each containing the 35S CaMV promoter (EC51288), the gene coding for the first 51 amino acids of the *Arabidopsis thaliana* F1-ATPase γ subunit (pFAγ51), *Arabidopsis thaliana* codon-optimized *nifH* (EC38011), and *N. benthamiana* codon-optimized *nifK* (EC38015), *nifY* (EC38019), *nifE* (EC38016), *nifN* (EC38024), *nifJ* (EC38022), *nifB* (EC38017), *nifQ* (EC38025), *nifF* (EC38021), *nifU* (EC38026), *nifS* (EC38018), *nifV* (EC38020), *nifW* (EC38027), *nifZ* (EC38029), *nifM* (EC38023), *nifX* (EC38028), *A. thaliana* codon-optimized HA epitope tag (EC38003), and CaMV terminator (EC41414) were assembled into plant expression backbone vectors containing left and right T-DNA borders (EC47772, EC47742, EC47751, EC47761, EC47781) using Type IIS restriction cloning. The plasmid ID and descriptions are listed in **Supplementary Table 1** and nucleotide sequences of the codon optimized *nif* genes in **Supplementary Material 2**.

### Plant Growth and Transient Transformation of *N. benthamiana*

*N. benthamiana* plants were grown in a Conviron growth chamber at 23°C under a 16:8 h light:dark cycle with 90 μmol/min light intensity provided by cool white fluorescent lamps. *Agrobacterium tumefaciens* strain GV3101 (SN vectors) or AGL1 (P19 vector) cells were grown to stationary phase at 28°C in LB broth supplemented with 50 mg/ml carbenicillin or 50 mg/ml kanamycin, according to the selectable marker gene on the vector, and 50 mg/ml rifampicin. Acetosyringone was added to the culture to a final concentration of 100 μM and the culture was then incubated for another 2.5 h at 28°C with shaking. The bacteria were pelleted by centrifugation at 5,000 x *g* for 10 min at room temperature. The supernatant was discarded, and the pellet was resuspended in 10 mM MES pH 5.7, 10 mM MgCl_2_ and 100 μM acetosyringone (infiltration buffer) after which the OD_600_ was measured. A volume of each culture, including the culture containing the viral suppressor construct 35S::P19, required to reach a final concentration of OD_600_ = 0.10 was added to a fresh tube. The final volume was made up with the infiltration buffer. Leaves of five-week-old plants were then infiltrated with the culture mixture and the plants were grown for five days after infiltration before leaf samples were harvested for further analysis/experiments.

### Western Blot Analysis of Nif Proteins Transiently Expressed in *N. benthamiana*

To assess the processing of mitochondrially targeted and cytosolically located proteins, leaf disks of 180 mm^2^ were harvested from *N. benthamiana* and the proteins were extracted, subjected to SDS-PAGE and western blot according to Allen et al. (2017). Monoclonal anti-HA antibody produced in mouse (Sigma-Aldrich) was used as the primary antibody (1:5,000 dilution) and Immun-Star Goat Anti-Mouse (GAM)-HRP conjugate (Bio-Rad) was used as the secondary antibody (1:5,000 dilution). The PageRuler™ Prestained Protein Ladder (Thermo Fisher Scientific) and the BenchMark™ Pre-Stained Protein Ladder (Thermo Fisher Scientific), which was re-calibrated against the unstained BenchMark™ protein ladder to 146 kDa, 91 kDa, 63 kDa, 50 kDa, 40 kDa, 33 kDa, 22 kDa, 17 kDa, 14 kDa, and 10 kDa, were used as molecular size markers.

For solubility testing the harvested leaf tissue was ground in liquid nitrogen using a mortar and pestle and transferred to a microfuge tube. Three hundred (300) μl of cold solubility buffer (50 mM Tris-HCl pH 8.0, 75 mM NaCl, 100 mM mannitol, 2 mM DTT, 0.5% (w/v) polyvinylpyrrolidone (average MW 40 kDa), 5% (v/v) glycerol, 0.2 mM PMSF, 10 μM leupeptin and 0.5% (v/v) Tween^®^ 20) was added and the samples were centrifuged for 5 min at 16,000 x g at 4°C. The supernatant was transferred to a fresh tube and the pellet was resuspended in 300 μl of fresh cold solubility buffer. Both, the supernatant (sample 1) and the resuspended pellet (sample 2) were centrifuged again for 5 min at 16,000 x *g* at 4°C. From sample 1 a subsample was taken, which is referred to as the soluble fraction. This subsample was mixed with an equivalent amount of 4 x SDS buffer (250 mM Tris-HCl pH 6.8, 8% (w/v) SDS, 40% (v/v) glycerol, 120 mM DTT, and 0.004% (w/v) bromophenol blue). After the second centrifugation step, the supernatant of sample 2 was discarded. The pellet is referred to as the insoluble fraction. The pellet was resuspended in 300 μl 4 x SDS buffer and 300 μl of solubility buffer were added. When soluble and insoluble fractions were compared to the amount of total protein, the leaf piece for the total protein sample was ground as described above. However, the ground sample was resuspended in 300 μl 4 x SDS buffer and 300 μl of solubility buffer were added. Samples for the total, insoluble and soluble fractions were heated at 95°C for 3 min and then centrifuged at 12,000 x *g* for 2 min. 20 μl of the supernatant containing the extracted polypeptides was loaded on a NuPAGE Bis Tris 4%–12% gels (Thermo Fisher Scientific) for gel electrophoresis and western blot analysis.

For western blot analysis of anaerobically extracted proteins, the extractions were carried out in an anaerobic chamber (COY Laboratory Products) filled with a H_2_/N_2_ atmosphere (2%–3%/97%–98%). Anaerobic extraction solutions were prepared at a Schlenk line in a bottle equipped with a butyl rubber septum by at least four cycles of evacuating and purging with N_2_. Leaf disks were ground in cold solubility buffer instead of liquid nitrogen.

### Isolation of pFAγ 51::NifU::Twin-Strep-Tag From *N. benthamiana*

*N. benthamiana* leaves infiltrated with pFAγ51::NifU::twin-Strep-tag^®^ (Schmidt et al., 2013) and P19 were harvested 4 days post infiltration. Three (3.0) g leaf tissue was ground in 30 ml of 100 mM Tris-HCl pH 8.0, 150 mM NaCl, 5% (v/v) glycerol, 2 mM TCEP, 1% (w/v) PVP (average MW 40 kDa), and 0.1% Tween 20 using a mortar and pestle. The extract was centrifuged at 40,000 x *g* for 30 min at 10°C. The supernatant was loaded on a StrepTactinXT (IBA Lifesciences) column with a column volume of 2 ml equilibrated in 100 mM Tris-HCl pH 8.0, 150 mM NaCl, 2 mM TCEP (wash buffer). After loading, the column was washed with 20 ml wash buffer and the protein was eluted with 5 ml 100 mM Tris-HCl pH 8.0, 150 mM NaCl, 2 mM TCEP, and 50 mM biotin. The eluate was concentrated using an Amicon^®^ Ultra centrifugal filter (10 kDa MWCO). Samples from the supernatant, flow through and eluate were subjected to SDS-PAGE on a 4%–12% NuPage SDS gel. Proteins were transferred to PVDF membranes with the iBlot dry blotting system (Thermo Fisher Scientific), washed with TBST and developed using the Strep-Tactin-HRP conjugate (IBA Lifesciences; 1:10,000 dilution). Assessment of protein loading was estimated by staining SDS-PAGE gels with SimplyBlue™ SafeStain (Thermo Fisher Scientific). The purified protein was subjected to trypsin digestion and proteomic analysis as outlined below.

### Construction of Modified MIT v2.1 Plasmids for Function Testing in *Escherichia coli*

First, MIT v2.1 (Smanski et al., 2014) was split into two parts for easier modification of the *nif* genes by PCR. The first half containing *nifHDKYENJ* was amplified with SbfI sites on either end (with oligos MIT_v2.1_SbfInifH_FW2 5’-*AACCTGCAGGTGACGTCTAAGAAAAGGAATATTCAGCAAT*-3’, and MIT_v2.1_SbfInifJ_RV2 5’-*AACCTGCAGGGCTAACTAACTAACCACGGACAAAAAACC*-3’) and ligated into pCR Blunt II TOPO (Thermo Fisher Scientific). The second half containing *nifBQFUSVWZM* was amplified with SbfI sites on either end (with oligos MIT_v2.1_SbfInifB_FW 5’- *AACCTGCAGGTACTCTAACCCCATCGGCCGTCTTA*-3’, and MIT_v2.1_SbfIori_RV 5’-*AACCTGCAGGTACGTAGCAATCAACTCACTGGCTC*-3’), digested with SbfI, and ligated back together. This religated plasmid, herein termed pB-ori, was used as a negative control for nitrogenase function testing. The positive control was constructed by ligating SbfI digested pCR Blunt II TOPO containing *nifHDKYENJ* and pB-ori. The scar9 extension (*ATGTCAACTCAAGTGGTGCGTAACCGC* coding for MSTQVVRNR) was added to the front of fw primers that bind to the start of the coding sequence for each *nif* gene, and rv primers were designed adjacent to the 5’ end of each *nif* gene that the scar9 was being added (primers listed in **Supplementary Table 5**). The amplified PCR product containing the scar9 extension in front of a given *nif* gene was ligated using ligation cycling reaction (LCR; de Kok et al., 2014). The other half of MIT v2.1 that was not modified was religated with the half with the scar9 extension *via* SbfI restriction sites. The plasmid ID and descriptions are listed in **Supplementary Table 2**.

### Acetylene Reduction Assay

Acetylene reduction assays on *E. coli* transformed with control plasmids or modified MIT v2.1, along with controller plasmid N249 (Temme et al., 2012) were carried out according to Dilworth (1966) with the following modifications: Transformed JM109 cells were grown aerobically overnight at 37°C in LB medium with antibiotics to OD_600 _= 1.0. The cultures were resuspended in induction medium (25 g/L Na_2_HPO_4_, 3 g/L KH_2_PO_4_, 0.25 g/L MgSO_4_.7H_2_O, 1 g/L NaCl, 0.1 g/L CaCl_2_.2H_2_O, 2.9 mg/L FeCl_3_, 0.25 mg/L Na_2_MoO_4_.2H_2_O, 20 g/L sucrose, 0.015% serine, 0.5% casamino acids, 5 mg/L biotin, 10 mg/L para-aminobenzoic acid, 1 mM isopropyl β-D-thiogalactopyranoside (IPTG), transferred to air-tight culture flasks, and headspace sparged with argon gas. After 5 h incubation at 30°C, 200 rpm, pure C_2_H_2_ was injected into the headspace at 10% (vol.) and incubated for a further 18 h. Production of ethylene was measured by gas chromatography with flame ionization detection (GC-FID) using a RT-Alumina Bond/MAPD column (30 m x 0.32 mm ID x 5 μm film thickness) with a 5 m particle trap column fitted to the detector end of an Agilent 6890N GC. Parameters for the GC-FID were as follows: the inlet and FID were set to 200°C, carrier gas (He) velocity at 35 cm/s, and isothermal oven temperature set to 120°C. The number of biological replicates of the assay for each JM109 containing control and modified MIT v2.1 plasmids ranged between 2 and 8, the details of which are provided in **Table 1**.

### *E. coli* Total Protein Extraction

IPTG-induced *E. coli* JM109 containing modified MIT v2.1 plasmid for the acetylene reduction assay as described above were used to extract protein with 8 M urea and 2% SDS in 100 mM Tris-HCl pH 8.5. Protein extracts were stored at -80°C prior to processing. Protein concentrations were estimated using the Bio-Rad microtiter Bradford protein assay (California, USA) according to the instructions provided (Bio-Rad version: Lit 33 Rev C) and measurements were made at 595 nm using a SpectraMax Plus. Bovine serum albumin (BSA) standard was used in the linear range 0.05 mg/ml to approximately 0.5 mg/ml. The BSA concentration was determined by high sensitivity amino acid analysis at Australian Proteomics Analysis Facility (Sydney, Australia).

### Tryptic Digestion of Protein for Proteomic Analysis

Protein was subjected to filter-aided sample preparation (Wiśniewski et al., 2011). In brief, 100 µl (~200 µg) of protein was diluted in 100 µl of 8 M urea, 100 mM Tris-HCl, pH 8.5 (UA buffer), and loaded onto a 10 kDa molecular weight cut-off (MWCO) centrifugal filter (Merck Millipore, Australia) and centrifuged at 20,800 x *g* for 15 min at 22˚C. The filter (and protein >10 kDa) was washed with 200 µl of UA buffer and centrifuged at 20,800 x *g* for 15 min at 22˚C. To reduce the protein on the filter, dithiothreitol (50 mM, 200 µl) was added and the solution incubated at room temperature for 50 min with shaking. The filter was washed with two 200 µl volumes of UA buffer with centrifugation (20,800 x *g*, 15 min). For cysteine alkylation, iodoacetamide (IAM) (100 µl, 50 mM IAM in UA buffer) was added and incubated in the dark for 30 min at 22˚C before centrifugation (20,800 x *g*, 15 min) and washed with two 200 µl volumes of UA buffer with centrifugation (20,800 x *g*, 15 min) followed by two subsequent wash/centrifugation steps with 200 µL of 50 mM ammonium bicarbonate. The trypsin (sequencing grade, Promega, Alexandria, Australia) solution (200 µl, 20 µg/ml (4 µg) in 50 mM ammonium bicarbonate and 1 mM CaCl_2_) was loaded onto the filter and incubated for 18 h at 37°C in a wet chamber. The tryptic peptides were collected by centrifugation (20,800 x *g*, 15 min) followed by an additional wash with 200 µl of 50 mM ammonium bicarbonate. The combined filtrates were lyophilized and stored at -20°C.

### Global Proteomic Profiling

The digested peptides were reconstituted in 50 µl of 1% formic acid (FA) and chromatographic separation (4 μl) on an Ekspert nanoLC415 (Eksigent, Dublin, CA, U.S.A.) directly coupled to a TripleTOF 6600 liquid chromatography tandem mass spectrometry (LC-MS/MS, SCIEX, Redwood City, CA, USA). The peptides were desalted for 5 min on a ChromXP C18 (3 μm, 120 Å, 10 mm × 0.3 mm) trap column at a flow rate of 10 μl/min 0.1% FA, and separated on a ChromXP C18 (3 μm, 120 Å, 150 mm × 0.3 mm) column at a flow rate of 5 μl/min at 30˚C. A linear gradient from 3-25% solvent B over 68 min was employed followed by: 5 min from 25% B to 35% B; 2 min 35% B to 80% B; 3 min at 80% B, 80-3% B, 1 min; and 8 min re-equilibration. The solvents were: (A) 5% dimethylsulfoxide (DMSO), 0.1% formic acid (FA), 94.9% water; (B) 5% DMSO, 0.1% FA, 90% acetonitrile, 4.9% water. The instrument parameters were: ion spray voltage 5,500 V, curtain gas 25 psi, GS1 15 psi, and GS2 15 psi, heated interface 150°C. Data were acquired in information-dependent acquisition mode comprising a time-of-flight (TOF)-MS survey scan followed by 30 MS/MS, each with a 40 ms accumulation time. First stage MS analysis was performed in positive ion mode, mass range *m/z* 400−1,250 and 0.25 s accumulation time. Tandem mass spectra were acquired on precursor ions >150 counts/s with charge state 2−5 and dynamic exclusion for 15 s with a 100 ppm mass tolerance. Spectra were acquired over the mass range of *m/z* 100−1,500 using the manufacturer’s rolling collision energy based on the size and charge of the precursor ion. Protein identification was undertaken using ProteinPilot™ 5.0 software (SCIEX) with searches conducted against the *E. coli* subset of the Uniprot-SwissProt database (2018/08) appended with a custom nitrogenase (Nif+Mit2Nif) database including the control chloramphenicol acetyltransferase (CAT/P62577) and a contaminant database (Common Repository of Adventitious Proteins). The total number of proteins in the custom database was 10820. The resulting mass spectrometry proteomics raw and result data have been deposited to the CSIRO public data access portal (https://doi.org/10.25919/5ea22a86a740c).

### Targeted Liquid Chromatography – Multiple Reaction Monitoring – Mass Spectrometry (LC-MRM-MS)

Reduced and alkylated tryptic peptides (5 μl) were chromatographically separated on a Kinetex C18 column (2.1 mm x 100 mm, Phenomenex) using a linear gradient of 5–45% acetonitrile (in 0.1% formic acid) over 10 min at a flow rate of 400 μl/min. The eluent from the Shimadzu Nexera UHPLC was directed to a QTRAP 6500 mass spectrometer (SCIEX) equipped with a TurboV ionization source operated in positive ion mode for data acquisition and analysis. The MS parameters were as follows: ion spray voltage, 5,500 V; curtain gas, 35; GS1, 35; GS2, 40; source temperature, 500°C; declustering potential, 70 V; and entrance potential, 10 V. Peptides were fragmented in the collision cell with nitrogen gas using rolling collision energy dependent on the size and charge on the size and charge of the precursor ion. Relative quantitation using scheduled multiple reaction monitoring (MRM) scanning experiments (MRM transition peptide information provided in **Supplementary Table 3**) with a 40 second detection window around the expected retention time (RT) and a 0.3 second cycle time. Data were acquired using Analyst v1.7 software. Peak areas of four MRM transitions were integrated using Skyline (MacLean, Bioinformatics 2010) wherein all transitions were required to co-elute with a signal-to-noise (S/N) > 3 and intensity >1,000 counts per second (cps) for detection.

### Identification of Proteotypic Peptides for NifM Protein in *E. coli*

From the identified peptides, two NifM peptides (DAFAPLAQR and DYLWQQSQQR) that were fully tryptic, contained no unusual cleavages and/or modifications and showed high response in the MS (as judged by peak intensity) were selected for multiple reaction monitoring scanning to confirm the detection of the nitrogenase (NifM) proteins in the *E. coli* JM109 expressions (**Supplementary. Table 3**). Each JM109 containing control and modified MIT v2.1 plasmids were analyzed once for presence of the nifM protein and presented in **Supplementary Figure 6**.

### Identification of Proteotypic Peptides for Chloramphenicol Acetyltransferase Protein in *E. coli*

Chloramphenicol acetyltransferase (CAT/P62577) enzyme is an effector of chloramphenicol resistance in bacteria and is expressed in all *E. coli* JM109 transformed with unmodified or modified MIT v2.1. This protein was selected as a control for protein expression. One peptide (four transitions/peptide) was selected to screen the expression of CAT (YYTQGDK, **Supplementary Table 3**). Each JM109 containing control and modified MIT v2.1 plasmids were analyzed once for presence of the CAT protein and presented in **Supplementary Figure 6**.

## Data Availability Statement

The raw data supporting the conclusions of this article will be made available by the authors, without undue reservation.

## Author Contributions

SO, CG, RA, and CW conceived the project and designed the experiments. SO, CG, RA, AM, DH, VG, EJ, KB, and MC conducted the experiments. All authors contributed to the article and approved the submitted version.

## Funding

This project was funded by CSIRO and Cotton Seed Distributors Pty Ltd. Cotton Seed Distributors Pty Ltd was not involved in the study design, collection, analysis, interpretation of data, the writing of this article or the decision to submit it for p​​ublication.

## Conflict of Interest

This work is subject to a patent application by CSIRO in which SO, CG, RA, and CW are inventors.

The remaining authors declare that the research was conducted in the absence of any commercial or financial relationships that could be construed as a potential conflict of interest.
