# Supplementary material for: A Synthetic Biology Workflow Reveals Variation in Processing and Solubility of Nitrogenase Proteins Targeted to Plant Mitochondria, and Differing Tolerance of Targeting Sequences in a Bacterial Nitrogenase Assay
Source: Front Plant Sci. 2020 Sep 10;11:552160. doi: 10.3389/fpls.2020.552160 (PMC7511584; doi:10.3389/fpls.2020.552160)
Supplement: Supplementary file 1 [file DataSheet_1.pdf]

Supplementary Material 1

Suppl. Table 1: Designations and descriptions of plasmids constructed to test mitochondrial targeting efficiency and protein solubility of Nif proteins transiently expressed in *Nicotiana benthamiana*. The protein size (kDa) was calculated using the ExPASy Compute pl/Mw tool with resolution set to monoisotopic (https://web.expasy.org/compute_pi/).

Plasmid ID	Description	calculated protein size (kDa)
SN192	pFAγ51::NifB::HA	58
SN38	pFAγ51::NifE::HA	57
SN138	pFAγ51::NifF::HA	26
SN27	pFAγ51::NifH::HA	39
SN139	pFAγ51::NifJ::HA	134
SN140	pFAγ51::HA::NifK	65
SN30	pFAγ51::NifM::HA	38
SN39	pFAγ51::NifN::HA	57
SN141	pFAγ51::NifQ::HA	27
SN31	pFAγ51::NifS::HA	50
SN32	pFAγ51::NifU::HA	36
SN142	pFAγ51::NifV::HA	48
SN143	pFAγ51::NifW::HA	17
SN144	pFAγ51::NifX::HA	26
SN145	pFAγ51::NifY::HA	32
SN146	pFAγ51::NifZ::HA	24
SN201	6×His::NifB::HA	53
SN203	6×His::NifE::HA	52
SN204	6×His::NifF::HA	21
SN205	6×His::NifH::HA	34
SN206	6×His::NifJ::HA	130
SN72	6×His::HA::NifK	61
SN207	6×His::NifM::HA	33
SN208	6×His::NifN::HA	53
SN209	6×His::NifQ::HA	22
SN210	6×His::NifS::HA	46
SN211	6×His::NifU::HA	32
SN212	6×His::NifV::HA	43
SN213	6×His::NifW::HA	12
SN214	6×His::NifX::HA	21
SN215	6×His::NifY::HA	27

SN216	6×His::NifZ::HA	19
SN166	pFAγ51::NifU::twin-Strep	38

Suppl. Table 2: Designations and descriptions of plasmids constructed for bacterial function testing in *Escherichia coli*.

Plasmid ID	Description
pSO003	MIT v2.1
pSO006	scar9::NifB
pSO009	scar9::NifD
pSO026	scar9::NifE
pSO032	scar9::NifF
pSO012	scar9::NifH
pSO028	scar9::NifJ
pSO029	scar9::NifK
pSO038	scar9::NifM
pSO027	scar9::NifN
pSO031	scar9::NifQ
pSO034	scar9::NifS
pSO033	scar9::NifU
pSO035	scar9::NifV
pSO036	scar9::NifW
pSO030	scar9::NifY
pSO037	scar9::NifZ
pSO051	Δ*nifM*
pSO013	NifK::HA
pSO004	pB-ori (Δ*nifHDKENJ*)

Suppl. Table 3: Multiple reaction monitoring transitions of chloramphenicol acyltransferase and NifM peptides for targeted liquid chromatography – multiple reaction monitoring – mass spectrometry. CAT, chloramphenicol acyltransferase.

**Protein**	**Peptide** [a]	**RT (min)** [b]	**Q1 *m/z*** [b]	**z** [b]	**Q3 *m/z*** [a]	**Fragment**	**CE** [c]
CAT	YYTQGDK	1.0	437.70	2+	319.16	y3+	20.45
					447.22	y4+	20.45
					548.26	y5+	20.45
					711.33	y6+	20.45
NifM-1	DAFAPLAQR	4.68	494.76	2+	584.35	y5+	23.24
					655.39	y6+	23.24
					802.46	y7+	23.24
					334.14	b3+	23.24
NifM-2	DYLWQQSQQR	4.47	676.32	2+	646.33	y5+	32.14
					774.39	y6+	32.14
					960.46	y7+	32.14
					279.10	b2+	32.14

a. The peptide sequence is represented by single amino acid code. C (cam) refers to carbamidomethyletion of cysteine.

b. RT, retention time (min); Q1 *m/z*, precursor ion mass-to-charge ratio; z, charge state; Q3 *m/z*, fragment ion *m/z*; CE, collision energy in V.

c. Collision energy settings were calculated (CE = slope (0.049) x (precursor *m/z*) + intercept (-1.0)) for a 6500 QTRAP mass spectrometer (AB SCIEX, Redwood City, USA).

Suppl. Table 4: Identification of *Klebsiella nif* gene homologues in the *Escherichia coli* K12 genome by tblastn analysis. NSH, no significant hit.

nif	Protein accession	% Coverage	% ID	*gene*	Product	UniProtKB/Swiss-Prot
H	NP_415693.1	27	42	*minD*	Z-ring positioning protein MinD	P0AEZ3
D		NSH				
K		NSH				
Y	NP_417649.1	14	39	*dacB*	peptidoglycan DD-endopeptidase DacB	P24228
E	NP_414655.1	8	38	*pdhR*	DNA-binding transcriptional dual regulator PdhR	P0ACL9
N	NP_415184.1	13	50	*rihA*	pyrimidine-specific ribonucleoside hydrolase rihA	P41409
J	NP_415896.1	99	48	*ydbK*	putative pyruvate-flavodoxin oxidoreductase	P52647
B	NP_416701.1	19	27	*ccmE*	periplasmic heme chaperone	P69490
Q		NSH				
F	NP_415210.1	97	43	*fldA*	flavodoxin 1	P61949
U	NP_417024.1	44	48	*iscU*	scaffold protein for iron-sulfur cluster assembly	P0ACD4
S	YP_026169.1	95	36	*iscS*	cysteine desulfurase	P0A6B7
V	NP_414616.1	96	31	*leuA*	2-isopropylmalate synthase	P09151
W		NSH				
Z	NP_417300.1	43	31	*ppdC*	putative YgdA	P08372
M	NP_414595.1	38	29	*surA*	peptidyl-prolyl cis-trans isomerase SurA	P0ABZ6

Suppl. Table 5: Primers used to add the scar9 peptide onto the N-terminus of each Nif protein via translational fusion. BO, bridging oligo.

Primer name	Primer sequence
scar9nifBfw	5’-ATGTCAACTCAAGTGGTGCGTAACCGCATGACCTCTTGTTCGTCGTT-3’
nifBbluntrv	5’-TTTAGCCCTCCTATGATTGATTTGATGTATTACAGAGAGG-3’
scar9nifB_BO	5’-GGTTACGCACCACTTGAGTTGACATTTTAGCCCTCCTATGATTGATTTGATG-3’
scar9nifDfw	5’-ATGTCAACTCAAGTGGTGCGTAACCGCATGATGACTAATGCTACTGGCGAACGTAAC-3’
nifDbluntrv	5’-CCGGCTCCTCCGCTAGATAAAAATGTGA-3’
scar9nifD_BO	5’-CGCACCACTTGAGTTGACATCCGGCTCCTCCGCTA-3’
scar9nifEfw	5’-ATGTCAACTCAAGTGGTGCGTAACCGCATGAAGGGTAACGAGATTCTTGCTCTGCTG-3’
nifEbluntrv	5’-TTGTAATAACCTCCAGTGATGAATTGAATAGTGTGG-3’
scar9nifE_BO	5’-GCGGTTACGCACCACTTGAGTTGACATTTGTAATAACCTCCAGTGATGAATTGAATAGTGTGGC-3’
scar9nifFfw	5’-ATGTCAACTCAAGTGGTGCGTAACCGCATGGCGAACATCGGCATCTTCTTTG-3’
nifFbluntrv	5’-GTAGTAAAGCCTCCTTATAATTGAGACTCTTGTCTC-3’
scar9nifF_BO	5’-GCGGTTACGCACCACTTGAGTTGACATGTAGTAAAGCCTCCTTATAATTGAGACTCTTGTCTCTCCC-3’
scar9nifHfw	5’-ATGTCAACTCAAGTGGTGCGTAACCGCATGACCATGCGTCAGTGC-3’
nifHbluntrv	5’-ATATGAAACCTCCTTAAATATATTATATATTTGTATCTCCCAATAGTGAGTCGTATTAGAGTCAC-3’
scar9nifH_BO	5’-GGTTACGCACCACTTGAGTTGACATATATGAAACCTCCTTAAATATATTATATATTTGTATCTCCC-3’
scar9nifJfw	5’-ATGTCAACTCAAGTGGTGCGTAACCGCATGAAAACTATGGACGGTAACGCTGCG-3’
nifJbluntrv	5’-GCTTAATTTCTCCATATTAATCTCTAGTTAATCCGCTGCG-3’
scar9nifJ_BO	5’-GTTTTCATGCGGTTACGCACCACTTGAGTTGACATGCTTAATTTCTCCATATTAATCTCTAGTTAATCCGCTGCGTACGCGC-3’
scar9nifKfw	5’-ATGTCAACTCAAGTGGTGCGTAACCGCATGTCTCAAACTATCGATAAAATCAACTCTTGTTACCCG-3’
nifKbluntrv	5’-GTTACCTCGCCTAATTTTTGAGAGTATGA-3’
scar9nifK_BO	5’-GCGGTTACGCACCACTTGAGTTGACATGTTACCTCGCCTAATTTTTGAGAGTATGAGATTGCAAG-3’
scar9nifMfw	5’-ATGTCAACTCAAGTGGTGCGTAACCGCATGAATCCGTGGCAGCGCTTTG-3’
nifMbluntrv	5’-TATAGACCTCCTGGGTAATAACTTCAGTCTCTG-3’
scar9nifM_BO	5’-GCGGTTACGCACCACTTGAGTTGACATTATAGACCTCCTGGGTAATAACTTCAGTCTCTGAGA-3’
scar9nifNfw	5’-ATGTCAACTCAAGTGGTGCGTAACCGCATGGCAGACATTTTCCGCACTGATAAGCC-3’
nifNbluntrv	5’-AATTACTTCTTCCAGGTGTGGTAGGTTTAGGTGC-3’
scar9nifN_BO	5’-GCGGTTACGCACCACTTGAGTTGACATAATTACTTCTTCCAGGTGTGGTAGGTTTAGGTGC-3’
scar9nifQfw	5’-ATGTCAACTCAAGTGGTGCGTAACCGCATGCCGCCATTGGACTGGTTGC-3’
nifQbluntrv	5’-GCTTAATTTCTCCTCTTTAATGCCACTACGTGC-3’
scar9nifQ_BO	5’-GCGGTTACGCACCACTTGAGTTGACATGCTTAATTTCTCCTCTTTAATGCCACTACGTGC-3’
scar9nifSfw	5’-ATGTCAACTCAAGTGGTGCGTAACCGCATGAAACAAGTGTACCTGGACAACAACG-3’
nifSbluntrv	5’-GGAAAACCTCCTTCGATTTCAGAATGGTC-3’
scar9nifS_BO	5’-GCGGTTACGCACCACTTGAGTTGACATGGAAAACCTCCTTCGATTTCAGAATGGTCTACG-3’
scar9nifUfw	5’-ATGTCAACTCAAGTGGTGCGTAACCGCATGTGGAACTACAGCGAGAAAGTCAAGG-3’
nifUbluntrv	5’-TAGGAACCTCCTTCGCTGGTTTATTTG-3’
scar9nifU_BO	5’-GCGGTTACGCACCACTTGAGTTGACATTAGGAACCTCCTTCGCTGGTTTATTTGTCAG-3’
scar9nifVfw	5’-ATGTCAACTCAAGTGGTGCGTAACCGCATGGAGCGCGTCTTGATCAACG-3’

nifVbluntrv	5’-ATGTTTCCTTGTGGCGAGTTAGGC-3’
scar9nifV_BO	5’-GCGGTTACGCACCACTTGAGTTGACATATGTTTCCTTGTGGCGAGTTAGGCT-3’
scar9nifWfw	5’-ATGTCAACTCAAGTGGTGCGTAACCGCATGGAGTGGTTTTACCAGATTCCGGG-3’
nifWbluntrv	5’-TCTGTTTCTACTCCCTTTCTCTTGAAACTATCG-3’
scar9nifW_BO	5’-GCGGTTACGCACCACTTGAGTTGACATTCTGTTTCTACTCCCTTTCTCTTGAAACTATCGGG-3’
scar9nifYfw	5’-ATGTCAACTCAAGTGGTGCGTAACCGCATGTCTGACAATGATACCCTGTTTTGGCG-3’
nifYbluntrv	5’-AGAAGTACCTCCGGGAGTGAGTATGG-3’
scar9nifY_BO	5’-GCGGTTACGCACCACTTGAGTTGACATAGAAGTACCTCCGGGAGTGAGTATGG-3’
scar9nifZfw	5’-ATGTCAACTCAAGTGGTGCGTAACCGCATGCGCCCGAAATTCACCTTCTC-3’
nifZbluntrv	5’-TGTATGACCTATATTGATTCGGGCTGGTG-3’
scar9nifZ_BO	5’-GCGGTTACGCACCACTTGAGTTGACATTGTATGACCTATATTGATTCGGGCTGGTGAAG-3’

Suppl. Fig. 1: Purification of NifU from plant leaves and sample preparation for proteomic analysis. **(A)** Coomassie stain of the supernatant, flow through and eluate from the StrepTactin purification. A contaminating band (*) was observed, most likely corresponding to Rubisco (large chain). **(B)** Western blot analysis of the same samples with a StrepTactin-HRP conjugate antibody. **(C)** Coomassie gel after excision of the NifU gel slice for proteomic analysis.

Suppl. Fig. 2: Assessment of MTP cleavage of pFAγ51::Nif::HA proteins. Whole blot images of the western blot analysis of individual pFAγ51::Nif::HA, pFAγ51::HA::NifK, 6×His::Nif::HA and 6×His::HA::NifK proteins transiently expressed in *Nicotiana benthamiana* leaf. C, cytosolic expression; M, mitochondrially targeted. Due to considerable variation in abundance of mitochondrially located proteins and cytosolic equivalents, mitochondrially located NifB, NifE, NifF, NifH, NifK, unprocessed NifM, NifN, NifU, NifV, NifW, NifX and NifZ in these images are overexposed. Cytosolic NifF and NifZ are also overexposed.

Suppl. Fig. 3: Whole blot images of the western blot analysis (α-HA) of individual pFAγ51::Nif::HA and pFAγ51::HA::NifK proteins transiently expressed in *N. benthamiana* leaf. T, total protein; I, insoluble fraction; S, soluble fraction.

Suppl. Fig. 4: Whole blot images of the western blot analysis (α-HA) of individual pFAγ51::Nif::HA and pFAγ51::HA::NifK proteins transiently expressed in *N. benthamiana* leaf. Proteins were extracted under anaerobic conditions. T, total protein; I, insoluble fraction; S, soluble fraction.

Suppl. Fig. 5: Whole blot images of the western blot analysis (α-HA) of pFAγ51::NifE::HA/pFAγ51::NifN::HA, and pFAγ51::NifH::HA/pFAγ51::NifM::HA proteins expressed individually or together in *N. benthamiana* leaf. T, total protein; I, insoluble fraction; S, soluble fraction.

Suppl. Fig. 6: Proteomic analysis of NifM and chloramphenicol acyltransferase abundance in *E. coli* with modified MIT v2.1 plasmids. Relative expression levels are presented as the sum of multiple reaction monitoring (MRM) peak areas of the three highest ion transitions of each peptide of the +2 charge state measured by liquid chromatography-tandem mass spectrometry. Pos, positive control unmodified MIT v2.1; B-ori, negative control containing *nifBQFUSVWZM*; Δ*nifM*, MIT v2.1 with nifM deleted. **(A)** MRM peak area of NifM peptide DAFAPLAQR (NifM-1) (n=1); **(B)** MRM peak

area of NifM peptide DYLWQQSQQR (NifM-2) (n=1); **(C)** MRM peak area of chloramphenicol acetyltransferase peptide YYTQGDK (CAT) (n=1).

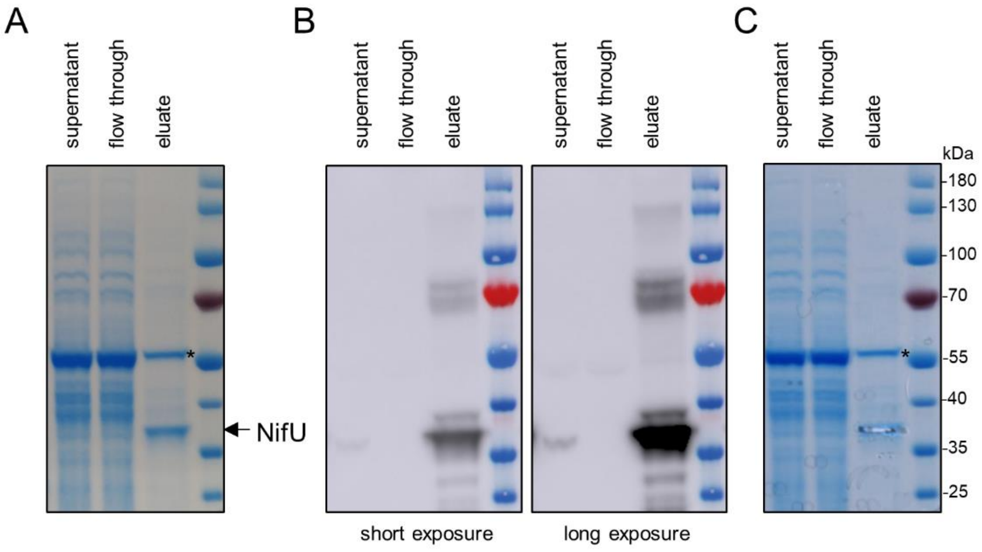
A
supernatant
flow through
eluate
*
NifU
B
supernatant
flow through
eluate
supernatant
flow through
eluate
short exposure
long exposure
C
supernatant
flow through
eluate
*
kDa
180
130
100
70
55
40
35
25

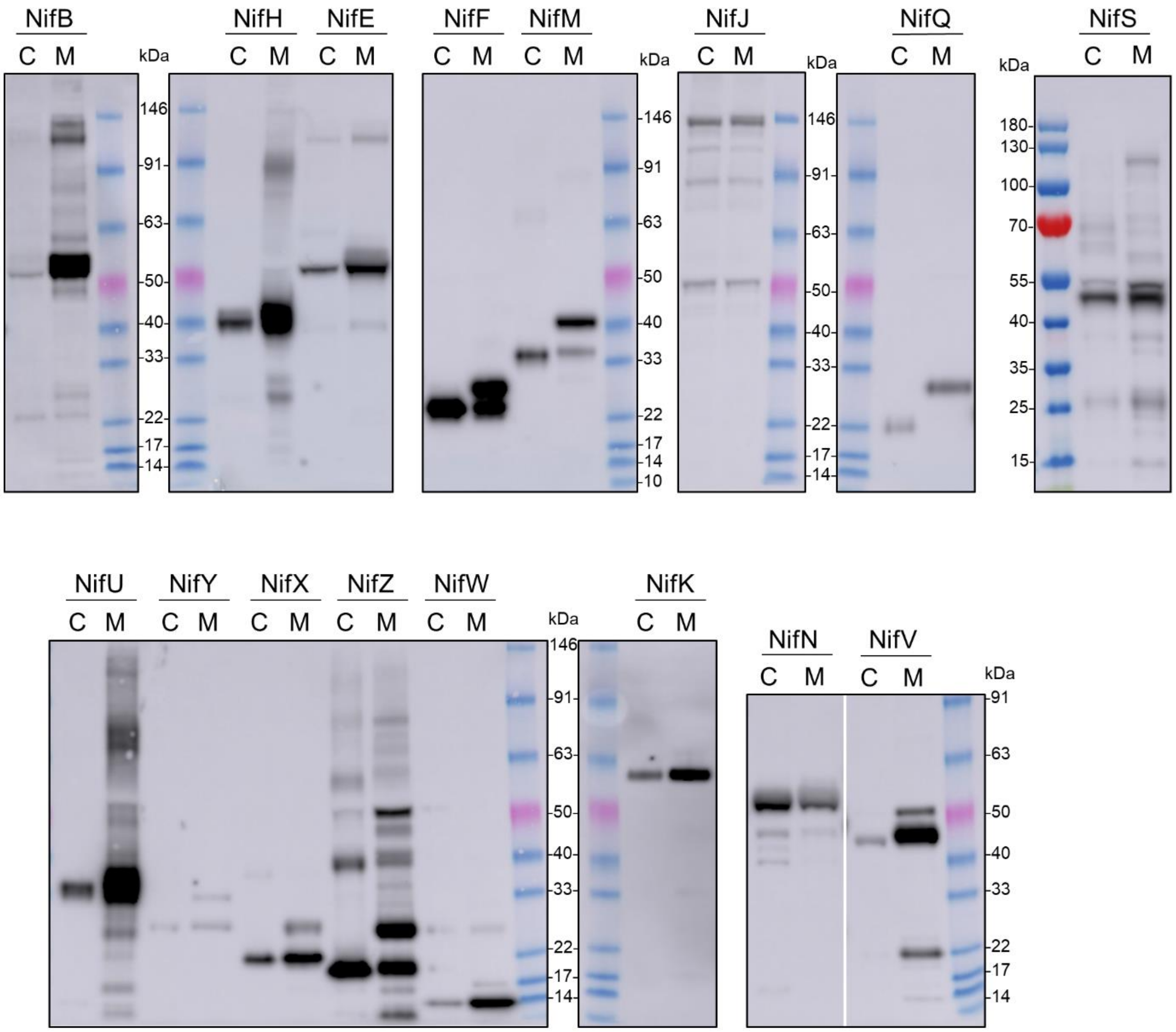
NifB
C M
kDa
146
91
63
50
40
33
22
17
14
NifH NifE
C M C M
NifF NifM
C M C M
kDa
146
91
63
50
40
33
22
17
14
10
NifJ
C M
kDa
146
91
63
50
40
33
22
17
14
NifQ
C M
kDa
180
130
100
70
55
40
35
25
15
NifS
C M
NifU NifY NifX NifZ NifW
C M C M C M C M C M
kDa
146
91
63
50
40
33
22
17
14
NifK
C M
NifN NifV
C M C M
kDa
91
63
50
40
33
22
17
14

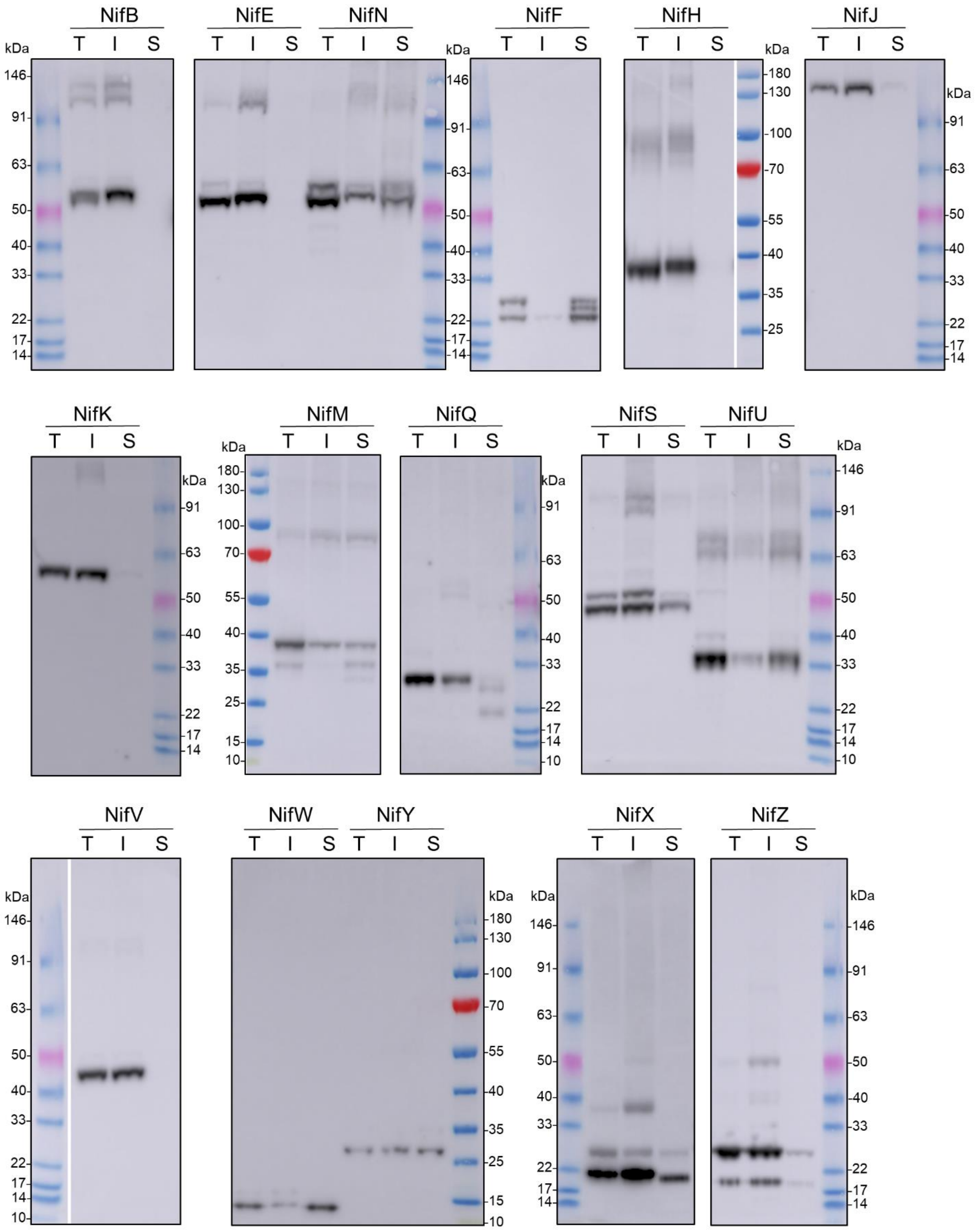
NifB
NifE
NifN
NifF
NifH
NifJ
NifK
NifM
NifQ
NifS
NifU
NifV
NifW
NifY
NifX
NifZ
T
I
S
kDa
146
91
63
50
40
33
22
17
14
180
130
100
70
55
35
25
15
10

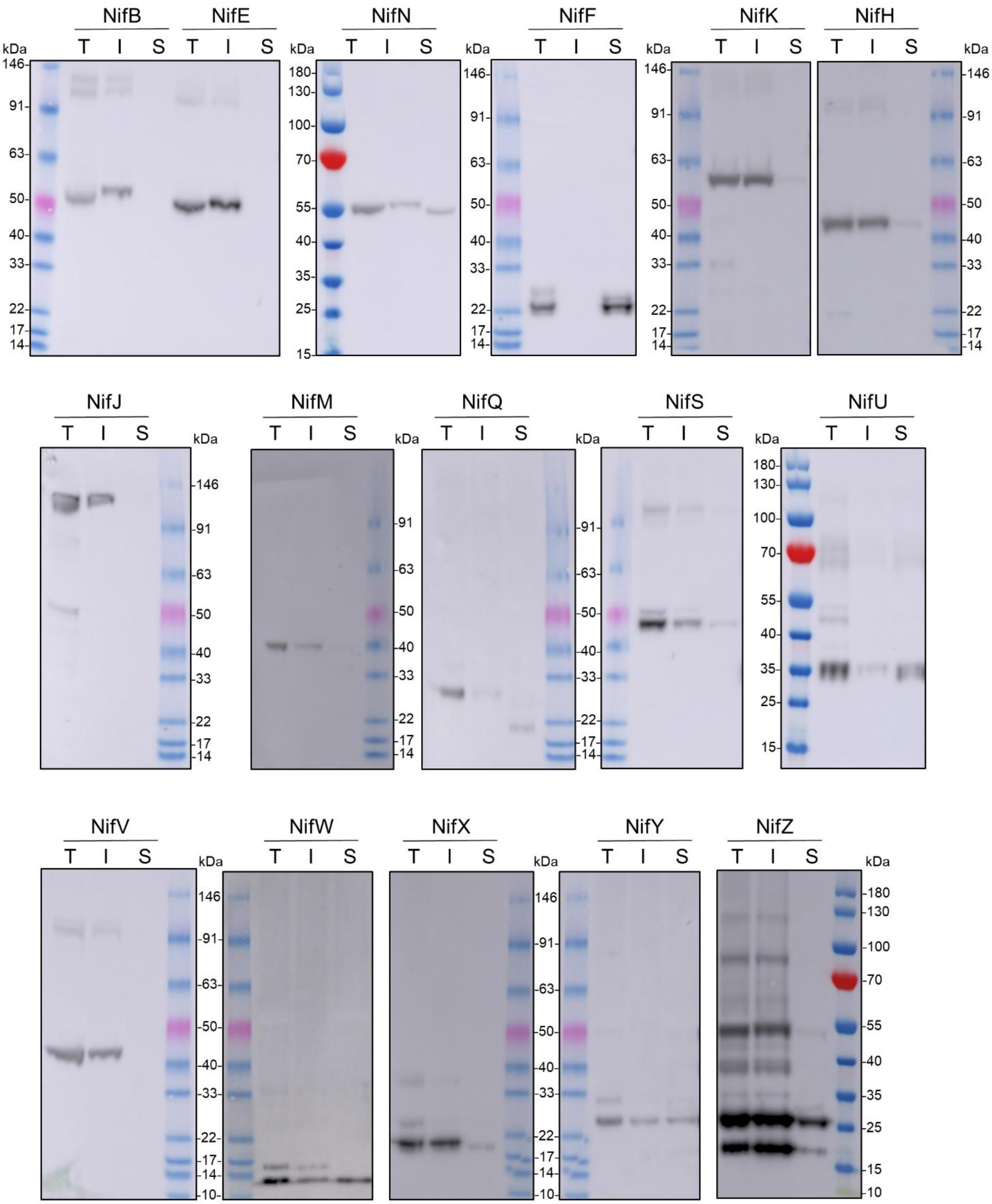
NifB
NifE
NifN
NifF
NifK
NifH
NifJ
NifM
NifQ
NifS
NifU
NifV
NifW
NifX
NifY
NifZ
T I S
kDa
180
146
130
100
91
70
63
55
50
40
35
33
25
22
17
15
14
10

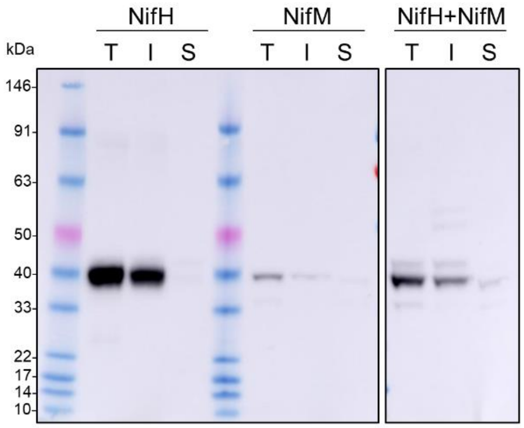
NifH
NifM
NifH+NifM
kDa
T I S T I S T I S
146
91
63
50
40
33
22
17
14
10

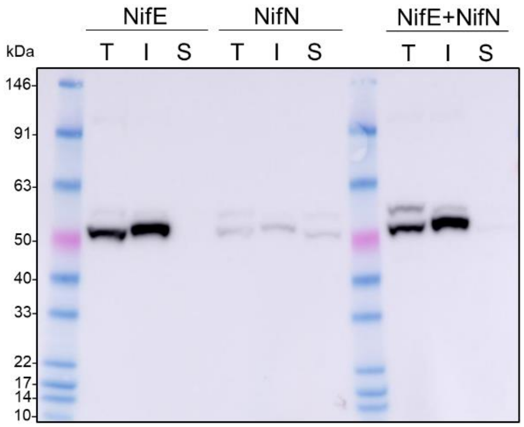
NifE
NifN
NifE+NifN
kDa
T I S T I S T I S
146
91
63
50
40
33
22
17
14
10

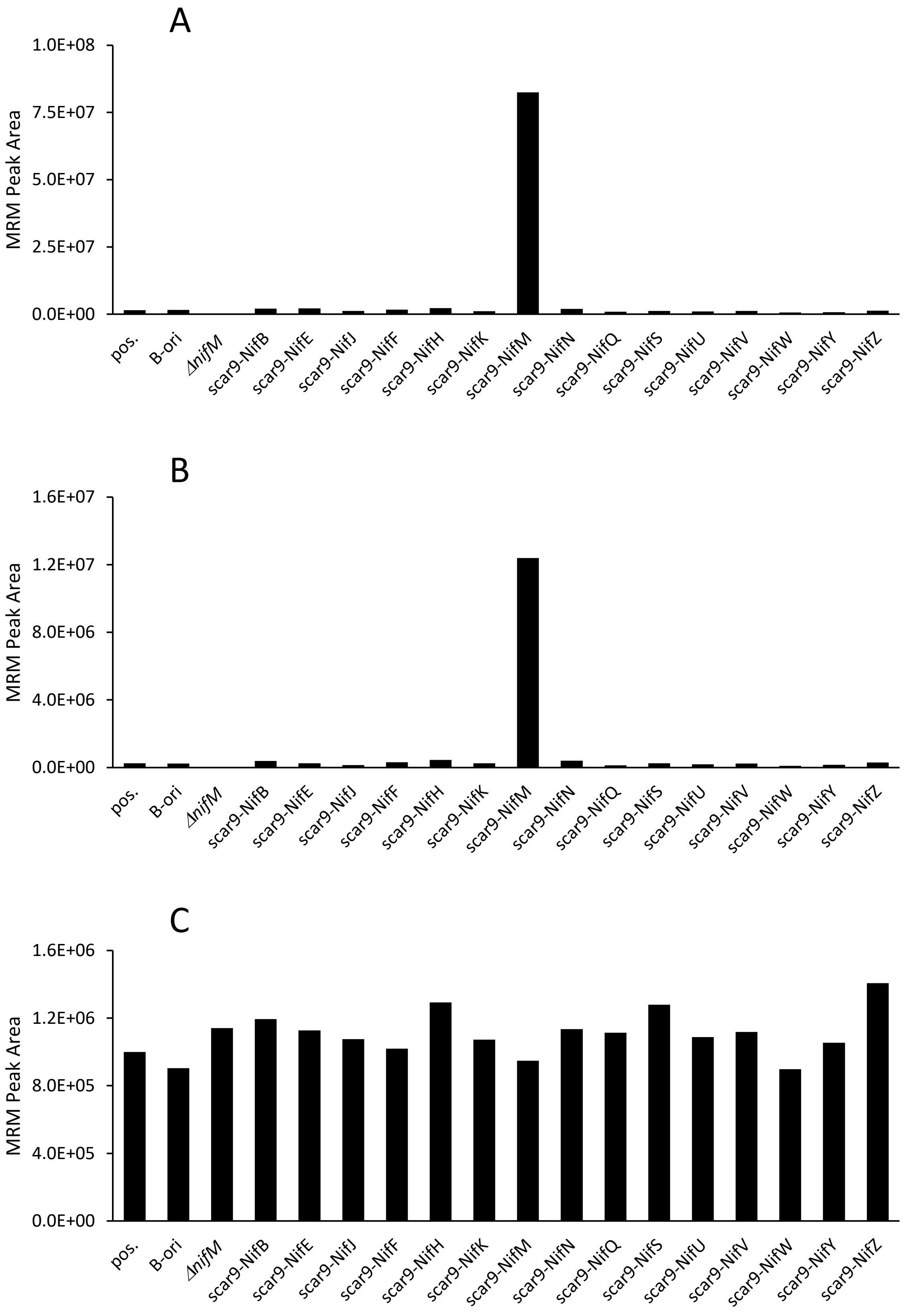
A
MRM Peak Area
1.0E+08
7.5E+07
5.0E+07
2.5E+07
0.0E+00
B
MRM Peak Area
1.6E+07
1.2E+07
8.0E+06
4.0E+06
0.0E+00
C
MRM Peak Area
1.6E+06
1.2E+06
8.0E+05
4.0E+05
0.0E+00
pos.
B-ori
ΔnifM
scar9-NifB
scar9-NifE
scar9-NifJ
scar9-NifF
scar9-NifH
scar9-NifK
scar9-NifM
scar9-NifN
scar9-NifQ
scar9-NifS
scar9-NifU
scar9-NifV
scar9-NifW
scar9-NifY
scar9-NifZ
