# Supplementary material for: A Synthetic Biology Workflow Reveals Variation in Processing and Solubility of Nitrogenase Proteins Targeted to Plant Mitochondria, and Differing Tolerance of Targeting Sequences in a Bacterial Nitrogenase Assay
Source: Front Plant Sci. 2020 Sep 10;11:552160. doi: 10.3389/fpls.2020.552160 (PMC7511584; doi:10.3389/fpls.2020.552160)
Supplement: Supplementary file 2 [file DataSheet_2.pdf]

Supplementary Material 2

Supplementary Material 2: Nucleotide sequences of *nif* genes used for *Nicotiana benthamiana* expression. SN designations are listed in Supplementary Material 1 Suppl. Table 1.

>nifB_SN192_SN201

ATGACATCATGCAGTTCTTTCTCAGGCGGAAAAGCATGTAGGCCCGCAGATGACTCAGC
ATTAACTCCTCTCGTAGCCGATAAAGCTGCCGCCCATCCATGTTATAGTAGGCACGGCCA
CCACCGTTTCGCCAGAATGCACCTTCCAGTAGCTCCAGCATGTAATTTACAATGCAACTA
CTGCAACAGGAAATTCGACTGCAGCAACGAATCTAGGCCTGGCGTAAGCTCTACTTTAC
TGACTCCTGAGCAAGCAGTGGTAAAGGTTAGACAAGTAGCTCAGGCCATACCACAATTG
AGTGTTGTCGGTATAGCCGGGCCAGGGGATCCATTAGCTAATATTGCACGTACATTTAG
AACTCTCGAGCTGATAAGAGAACAATTGCCGGATTTAAAACTGTGCCTCAGTACGAACG
GACTGATGCTGCCGGACGCAGTTGACAGATTGCTTGATGTTGGTGTTGACCATGTCACA
GTAACAATCAATACGTTGGACGCAGAAATTGCTGCTCAGATATACGCTTGGCTGTGGTT
GGATGGGGAGAGGTATTCCGGCAGGGAGGCCGGTGAAATATTAATCGCTAGGCAGCTG
GAGGGCGTTAGACGTCTGACTGCAAAGGGAGTCCTTGTTAAAATCAATAGCGTCCTTAT
TCCTGGCATCAACGATAGCGGTATGGCTGATGTAAGCAGAGCCTTGCGTGCTTCCGGGG
CATTCATTCATAACATTATGCCTTTAATCGCACGTCCAGAACACGGGACGGTTTTTGGGC
TCAACGGGCAGCCTGAGCCGGACGCTGAAACGCTGGCTGCCACCAGAAGCAGGTGCGG
CGAAGTAATGCCGCAGATGACACATTGTCACCAATGCCGAGCCGATGCCATTGGCATGT
TGGGTGAGGACCGAAGTCAACAATTCACCCAGCTGCCAGCTCCCGAGAGCCTTCCGGCC
TGGCTTCCAATATTGCATCAAAGAGCACAACTGCATGCTTCAATCGCAACCCGAGGCGA
ATCTGAAGCCGACGACGCTTGCTTGGTTGCTGTGGCAAGTAGCAGGGGTGACGTTATAG
ACTGTCATTTTGGTCACGCAGACAGATTCTATATTTATAGTTTGTCCGCAGCTGGCATGG
TTTTGGTAAATGAAAGATTTACCCCAAAGTATTGCCAAGGTCGAGATGACTGTGAGCCT
CAGGATAACGCAGCCAGATTTGCTGCTATTCTCGAGTTGCTGGCAGATGTGAAGGCCGT
ATTTTGTGTGAGAATCGGCCACACGCCCTGGCAGCAGTTGGAGCAAGAGGGCATCGAAC
CTTGTGTGGACGGAGCATGGAGGCCCGTCAGTGAGGTCTTGCCTGCATGGTGGCAGCAA
AGACGTGGAAGTTGGCCTGCTGCACTCCCCCACAAAGGGGTTGCT

>nifE_SN38_SN203

ATGAAGGGCAACGAGATTCTGGCTCTGCTTGATGAACCTGCTTGCGAGCATAACCACAA
GCAGAAGTCTGGTTGCTCTGCTCCTAAGCCTGGTGCTACTGCTGGTGGTTGTGCTTTTGA
TGGTGCTCAGATTACCCTGCTGCCTATCGCTGATGTTGCTCATCTTGTGCACGGTCCTATT
GGTTGCGCTGGTTCTTCTTGGGATAACAGGGGTTCTGCTAGCTCTGGTCCTACCTTGAAT
AGGCTTGGTTTCACCACCGATCTGAACGAGCAGGATGTGATTATGGGTAGGGGTGAGAG
AAGGCTTTTCCATGCTGTGAGGCATATCGTGACCAGGTATCATCCTGCTGCCGTGTTCAT
CTACAATACCTGCGTTCCAGCTATGGAAGGCGACGATCTTGAAGCTGTTTGTCAGGCTGC
TCAGACTGCTACTGGTGTTCCTGTGATTGCTATCGATGCTGCTGGTTTCTACGGCAGCAA
GAACCTTGGTAATAGGCTGGCTGGTGACGTGATGGTGAAGAGAGTTATTGGTCAGAGAG
AGCCTGCTCCTTGGCCTGAGTCTACTCTTTTTGCTCCTGAGCAGAGGCACGACATTGGTC
TTATTGGCGAGTTCAACATTGCCGGCGAGTTCTGGCATATTCAGCCTTTGTTGGATGAGC

TGGGCATCAGAGTGCTTGGATCTTTGTCTGGTGATGGCAGGTTCGCTGAGATTCAGACA
ATGCATAGGGCCCAAGCTAACATGCTTGTGTGCTCTAGGGCTCTGATCAATGTGGCTAG
AGCTTTGGAACAGAGGTACGGAACTCCTTGGTTTGAGGGCTCTTTCTACGGTATCAGGG
CTACCTCTGATGCTCTTAGACAGCTTGCTGCTCTGCTGGGTGATGATGATCTTAGGCAGA
GAACCGAGGCTCTTATCGCTAGAGAAGAACAGGCTGCTGAGCTTGCACTTCAGCCTTGG
AGAGAACAGCTTAGAGGTAGGAAGGCTCTCCTTTACACTGGTGGTGTGAAGTCTTGGTC
TGTGGTTTCTGCTCTGCAGGATCTTGGTATGACTGTGGTGGCTACTGGCACTAGGAAGTC
TACCGAAGAGGATAAGCAGAGGATCCGTGAGCTTATGGGTGAAGAGGCTGTGATGCTTG
AAGAGGGTAATGCTAGGACCCTTCTGGATGTGGTGTACAGGTATCAGGCTGACCTTATG
ATCGCTGGCGGTAGGAATATGTACACCGCTTACAAGGCTAGGCTGCCGTTCCTGGACAT
CAATCAAGAAAGAGAGCACGCTTTCGCTGGCTACCAGGGTATTGTGACTCTTGCTAGAC
AGCTGTGCCAGACCATCAACTCTCCAATTTGGCCTCAGACTCACTCTAGAGCACCTTGGA
GA

>nifF_SN138_SN204

ATGGCTAACATCGGCATTTTCTTCGGCACCGATACCGGCAAGACCCGGAAGATTGCTAA
GATGATTCACAAGCAGCTGGGCGAGCTTGCTGATGCTCCTGTGAATATCAACAGGACCA
CCTTGGACGACTTTATGGCTTACCCTGTGCTTCTTCTTGGCACCCCTACTCTTGGTGATGG
TCAACTTCCTGGTCTTGAGGCTGGTTGTGAGTCTGAATCTTGGAGCGAGTTCATCAGCGG
TCTGGATGATGCTTCTCTGAAGGGTAAGACCGTGGCTCTTTTCGGTCTTGGAGATCAGAG
GGGTTACCCTGACAATTTCGTGTCTGGTATGAGGCCTCTGTTCGACGCTCTTTCTGCTAG
AGGTGCTCAGATGATTGGCTCTTGGCCTAATGAGGGCTACGAGTTCTCTGCTTCTTCAGC
TCTTGAGGGCGATAGATTCGTGGGTCTTGTGCTGGATCAGGACAACCAGTTTGATCAGA
CTGAGGCTAGGCTGGCTTCTTGGCTTGAAGAGATTAAGAGGACCGTGCTT

>nifH_SN27_SN205

ATGACCATGCGTCAGTGTGCCATTTACGGTAAGGGCGGTATTGGCAAATCAACTACTAC
CCAAAACTTGGTAGCCGCTTTAGCTGAGATGGGCAAAAAGGTGATGATTGTGGGGTGCG
ATCCTAAAGCGGATTCAACTAGGCTTATTTTACATGCTAAGGCGCAAAATACAATTATG
GAGATGGCCGCCGAGGTGGGTAGCGTTGAGGACTTAGAGCTCGAGGACGTTTTGCAGAT
CGGTTACGGGGACGTTAGGTGCGCCGAATCTGGAGGGCCTGAGCCCGGCGTCGGTTGCG
CGGGCAGGGGTGTGATTACGGCGATCAACTTTCTTGAAGAAGAGGGGGCATACGAGGA
TGACCTCGACTTCGTATTTTATGATGTATTAGGCGATGTCGTGTGCGGGGGATTTGCAAT
GCCTATCAGGGAAAACAAGGCTCAAGAGATTTACATTGTATGCTCTGGTGAAATGATGG
CGATGTACGCGGCGAACAATATCTCCAAGGGCATCGTTAAATATGCAAAGTCTGGGAAA
GTAAGGCTAGGGGGCCTTATATGTAACTCAAGGCAGACAGACAGGGAAGATGAGCTCA
TTATTGCCTTGGCGGAGAAACTCGGTACGCAAATGATCCATTTCGTACCGAGGGACAAT
ATAGTTCAACGTGCAGAGATTAGACGTATGACCGTAATCGAGTATGACCCTGCTTGCAA
ACAAGCCAATGAATATAGGACTCTTGCCCAGAAAATAGTCAACAATACTATGAAAGTAG
TACCAACCCCGTGTACTATGGATGAGCTTGAAAGTTTATTAATGGAGTTTGGGATTATGG
AAGAGGAAGATACGTCCATAATCGGCAAGACAGCGGCAGAAGAAAATGCCGCG

>nifJ_SN139_SN206

ATGAGCGGCAAGATGAAAACCATGGATGGTAACGCTGCTGCCGCCTGGATTTCTTACGC
TTTCACTGAAGTGGCTGCTATCTACCCGATCACTCCTTCTACTCCTATGGCTGAGAATGT

GGATGAATGGGCTGCTCAGGGTAAGAAGAACCTTTTCGGTCAGCCTGTGCGGCTGATGG
AAATGCAATCTGAGGCTGGTGCTGCTGGTGCAGTTCATGGTGCACTTCAAGCTGGTGCTC
TTACTACTACCTATACCGCTTCTCAGGGTCTGCTGCTGATGATCCCTAACATGTACAAGA
TCGCTGGTGAGCTTCTGCCTGGTGTGTTCCATGTTTCTGCTAGGGCTCTTGCTACCAACA
GCCTGAACATTTTCGGCGATCACCAGGATGTTATGGCTGTGAGACAAACCGGTTGCGCT
ATGCTTGCTGAGAACAATGTGCAGCAGGTCATGGATCTTTCTGCTGTGGCTCACCTTGCT
GCTATCAAGGGTAGAATCCCGTTCGTGAACTTCTTCGATGGCTTCAGGACCTCTCACGAG
ATCCAGAAGATTGAGGTCTTGGAGTACGAGCAGCTTGCTACCCTTCTTGATAGGCCTGCT
CTGGATTCTTTCAGGCGGAATGCACTTCATCCTGACCATCCTGTGATTAGGGGTACTGCT
CAGAACCCGGACATCTACTTTCAAGAGAGAGAGGCTGGCAACAGGTTCTACCAAGCTCT
GCCTGATATCGTCGAGAGCTACATGACCCAGATTTCTGCTCTTACCGGCAGAGAGTACC
ACCTGTTCAATTACACTGGTGCCGCTGATGCTGAGCGGGTGATCATTGCTATGGGTTCTG
TTTGCGACACCGTGCAAGAGGTGGTGGATACTCTTAATGCTGCTGGCGAGAAGGTGGGC
CTTCTTTCTGTTCATCTTTTCCGGCCTTTCAGCCTGGCTCATTTCTTTGCTCAGTTGCCTAA
GACCGTGCAGAGGATTGCTGTGCTGGATAGGACAAAAGAACCTGGTGCTCAGGCTGAGC
CTCTTTGCCTTGATGTGAAGAACGCTTTCTACCACCACGATGACGCTCCTCTTATCGTTG
GTGGAAGGTATGCTCTTGGTGGCAAGGATGTGCTGCCTAACGATATTGCTGCTGTGTTCG
ACAACCTGAACAAGCCTCTTCCTATGGACGGTTTCACCCTGGGTATTGTGGATGACGTGA
CCTTCACTTCTTTGCCTCCAAGGCAACAGACCCTTGCTGTGTCTCATGATGGTATCACCG
CTTGCAAGTTCTGGGGTATGGGATCTGATGGTACTGTGGGCGCTAACAAGAGCGCCATT
AAGATCATCGGTGACAAGACCCCACTTTACGCTCAGGCCTACTTCAGCTACGACTCTAA
GAAGTCTGGCGGCATTACCGTGTCTCACCTTAGGTTTGGTGACCGGCCTATTAACAGCCC
TTACCTTATTCACCGGGCCGACTTCATTTCATGCAGCCAGCAGTCTTACGTCGAGAGGTA
CGATCTTCTCGATGGTCTTAAGCCTGGTGGCACCTTCCTTTTGAACTGCTCATGGTCAGA
TGCCGAGCTTGAGCAGCATCTTCCTGTGGGATTCAAGAGATACCTGGCTCGTGAGAACA
TCCACTTCTACACCCTTAACGCTGTGGATATCGCACGTGAGCTTGGTCTTGGTGGTAGGT
TCAACATGCTTATGCAGGCCGCCTTCTTCAAGCTTGCTGCAATTATTGATCCTCAGACCG
CTGCCGATTACCTGAAGCAAGCTGTTGAGAAGTCCTACGGTTCTAAGGGCGCTGCTGTC
ATTGAGATGAACCAGAGGGCTATTGAGCTTGGCATGGCTTCTCTGCATCAGGTGACAAT
TCCAGCTCATTGGGCTACCTTGGATGAGCCTGCAGCTCAAGCTTCTGCTATGATGCCTGA
TTTCATCCGGGATATCCTGCAGCCTATGAATAGGCAGTGTGGTGATCAGCTTCCTGTGAG
CGCTTTCGTCGGTATGGAAGATGGTACTTTCCCTTCTGGTACTGCTGCTTGGGAGAAGAG
GGGTATTGCTCTTGAAGTTCCTGTTTGGCAGCCTGAGGGTTGCACTCAATGTAATCAGTG
CGCTTTCATCTGCCCTCACGCTGCTATTAGACCTGCTCTTTTGAACGGCGAGGAACATGA
TGCAGCTCCAGTGGGTTTGCTTTCTAAGCCTGCTCAAGGCGCCAAAGAGTACCATTACCA
CCTTGCTATTAGCCCGCTGGATTGCTCTGGTTGCGGTAATTGTGTGGACATCTGTCCTGC
TAGAGGCAAGGCTCTTAAGATGCAGAGCCTTGATTCTCAGAGGCAGATGGCTCCAGTTT
GGGATTACGCTCTTGCTCTGACCCCTAAGAGCAACCCTTTCAGAAAGACTACCGTGAAG
GGCAGCCAGTTCGAGACTCCTTTGCTTGAGTTTTCTGGTGCTTGCGCTGGTTGTGGTGAG
ACTCCATATGCTAGGCTTATCACCCAGCTGTTCGGTGACAGGATGCTTATTGCTAACGCT
ACCGGCTGCAGCTCTATTTGGGGTGCTAGTGCTCCTTCTATCCCGTACACTACTAACCAC
AGAGGTCATGGTCCTGCTTGGGCTAACTCTCTGTTCGAGGATAATGCTGAGTTCGGCCTG
GGTATGATGCTTGGTGGACAGGCTGTTAGACAGCAGATCGCTGATGATATGACCGCTGC
TCTTGCTTTGCCTGTGTCTGATGAGCTGTCTGATGCTATGAGACAGTGGCTGGCTAAGCA
GGACGAAGGTGAAGGTACTAGAGAAAGGGCTGATAGGCTTAGCGAGAGACTGGCTGCT
GAGAAAGAGGGTGTTCCACTTCTTGAACAGCTGTGGCAGAACAGGGACTACTTCGTTAG
AAGGTCCCAGTGGATCTTCGGTGGTGATGGTTGGGCTTACGATATCGGTTTCGGTGGTCT
TGATCACGTGTTGGCTAGTGGTGAGGATGTGAACATTCTGGTGTTCGATACCGAGGTGT

ACAGCAACACTGGTGGCCAGTCTAGTAAGTCTACTCCAGTGGCTGCAATCGCTAAGTTT
GCTGCACAAGGTAAGCGGACCCGGAAGAAAGATCTTGGCATGATGGCTATGAGCTACG
GCAATGTGTATGTGGCTCAGGTTGCAATGGGCGCTGATAAGGATCAGACCCTTAGAGCT
ATTGCTGAGGCTGAAGCTTGGCCTGGTCCTTCTCTTGTGATTGCTTATGCTGCCTGCATC
AACCACGGTCTGAAGGCTGGTATGAGATGCTCTCAGAGAGAAGCTAAGAGGGCAGTTG
AGGCTGGATACTGGCATCTTTGGAGGTATCATCCACAGAGGGAAGCTGAGGGTAAGACC
CCTTTCATGCTGGATTCTGAGGAACCGGAAGAGAGCTTCAGGGATTTCTTGCTTGGTGAG
GTGAGGTACGCTAGCCTTCATAAGACTACCCCTCACTTGGCTGACGCTTTGTTCTCTAGG
ACTGAAGAGGATGCTCGTGCTAGGTTCGCTCAGTATAGAAGGCTTGCTGGTGAAGAG

>nifK_SN140_SN72

ATGAGCCAGACCATCGACAAGATCAACTCTTGCTACCCTCTGTTCGAGCAGGACGAGTA
CCAAGAGCTGTTTAGGAACAAGAGGCAGCTGGAAGAGGCTCATGATGCTCAGAGGGTT
CAAGAGGTTTTCGCTTGGACTACTACCGCTGAGTACGAGGCTCTTAACTTCCAGAGGGA
AGCTCTTACTGTGGACCCTGCTAAGGCTTGTCAACCTCTTGGTGCTGTGCTTTGCTCTCTG
GGTTTCGCTAACACCCTTCCTTACGTTCACGGTTCTCAGGGTTGTGTGGCTTACTTCAGG
ACCTACTTCAACCGGCACTTCAAAGAACCTATCGCCTGCGTGTCAGACAGCATGACTGA
AGATGCTGCTGTGTTCGGTGGCAACAACAACATGAACCTTGGTCTGCAGAACGCTAGCG
CACTTTACAAGCCTGAGATTATCGCTGTGAGCACTACCTGCATGGCTGAGGTTATCGGTG
ATGATCTGCAGGCCTTCATTGCCAACGCTAAGAAGGATGGCTTCGTCGACTCTAGCATTG
CTGTGCCTCATGCTCATACCCCAAGCTTCATTGGTTCTCATGTGACCGGCTGGGACAACA
TGTTTGAGGGATTCGCTAAGACCTTCACCGCTGATTATCAAGGTCAGCCTGGTAAGCTGC
CTAAGCTTAATCTTGTGACCGGGTTCGAGACTTACCTGGGCAATTTCAGGGTGCTGAAGC
GGATGATGGAACAGATGGCTGTTCCTTGCAGCCTCCTGTCTGATCCTTCTGAGGTTTTGG
ATACCCCTGCTGATGGTCACTACAGGATGTACTCTGGTGGTACTACCCAGCAAGAGATG
AAGGAAGCTCCTGACGCTATTGATACCCTTCTGCTTCAACCTTGGCAGCTGCTGAAGTCC
AAGAAAGTGGTGCAAGAAATGTGGAACCAGCCGGCTACTGAGGTGGCAATTCCTCTTGG
TCTTGCTGCTACCGATGAGCTTCTTATGACCGTGTCTCAGCTGTCCGGTAAGCCTATTGC
TGATGCTCTTACTCTTGAGAGGGGCAGACTGGTGGATATGATGCTTGATTCTCACACCTG
GCTGCACGGCAAGAAATTCGGTCTTTACGGCGATCCTGATTTCGTGATGGGTCTGACCA
GATTCCTGCTTGAGCTTGGTTGTGAGCCTACCGTGATTCTGTCTCACAACGCTAACAAGC
GGTGGCAAAAGGCCATGAACAAGATGCTGGATGCTAGCCCTTACGGCAGGGATAGTGA
GGTGTTCATTAACTGCGATCTGTGGCACTTCCGGTCCTTGATGTTTACTAGGCAGCCGGA
TTTCATGATCGGCAACAGCTACGGTAAGTTCATCCAGCGGGATACCCTGGCTAAGGGCA
AAGCTTTTGAGGTGCCACTTATCAGGCTTGGCTTCCCACTTTTCGATAGGCATCATCTTC
ATCGGCAGACCACTTGGGGTTACGAGGGTGCTATGAACATTGTGACCACCTTGGTGAAC
GCCGTGCTTGAGAAGTTGGATAGCGATACCTCTCAGCTGGGCAAGACCGATTACTCTTTC
GATCTTGTGCGGTAG

>nifM_SN30_SN207

ATGAATCCGTGGCAGAGATTTGCTAGGCAGAGGCTTGCTAGGTCTAGGTGGAATAGAGA
TCCTGCTGCTCTTGACCCTGCTGATACTCCTGCTTTTGAACAGGCTTGGCAGAGGCAGTG
TCATATGGAACAGACTATCGTTGCTAGGGTGCCAGAGGGTGATATTCCAGCTGCTCTGCT
TGAGAACATTGCTGCTTCTCTTGCTATCTGGCTGGATGAGGGCGATTTTGCTCCTCCTGA
AAGGGCTGCTATTGTGAGGCATCATGCTAGGCTTGAGCTGGCTTTCGCTGATATTGCTAG
ACAAGCTCCTCAGCCTGATCTGTCTACTGTGCAGGCTTGGTATCTTAGGCATCAGACCCA

GTTTATGAGGCCTGAGCAGAGGTTGACTAGGCACCTTCTTTTGACCGTGGATAACGATA
GGGAAGCTGTGCACCAGAGGATTCTTGGTCTTTACAGGCAGATCAACGCTAGCAGGGAT
GCTTTTGCTCCACTTGCTCAGAGGCATTCTCATTGCCCTTCAGCTCTTGAGGAAGGTAGG
CTTGGTTGGATTTCTAGGGGTCTGCTTTACCCGCAGCTTGAGACTGCTCTTTTCAGCCTTG
CTGAGAACGCTCTGTCTCTGCCTATTGCTTCTGAGCTTGGCTGGCATCTTCTTTGGTGCGA
GGCTATTAGACCTGCCGCTCCTATGGAACCTCAACAGGCTCTTGAAAGCGCTAGGGATT
ATCTGTGGCAACAGTCTCAGCAGAGACACCAGAGACAATGGCTTGAGCAGATGATCTCT
AGGCAGCCTGGTCTTTGTGGT

>nifN_SN39_SN208

ATGGCTGACATCTTCAGGACCGATAAGCCTCTTGCTGTGAGCCCTATTAAGACCGGTCA
ACCTCTTGGTGCTATCCTGGCTTCTCTTGGTATCGAGCACTCTATTCCTCTTGTGCATGGT
GCTCAGGGATGCAGCGCTTTTGCTAAGGTGTTCTTCATCCAGCACTTCCACGATCCTGTG
CCTCTTCAGTCTACTGCTATGGATCCTACCAGCACCATCATGGGTGCTGATGGTAACATC
TTTACCGCTCTGGATACCCTGTGCCAGAGGAACAATCCTCAGGCTATTGTGCTTCTGAGC
ACCGGTCTTTCTGAAGCTCAGGGCTCTGATATCTCTAGGGTTGTGAGGCAGTTCCGGGAA
GAGTATCCTAGGCATAAGGGTGTCGCTATCCTTACCGTGAACACCCCTGATTTCTACGGC
AGCATGGAAAACGGTTTCAGCGCTGTTCTTGAGAGCGTTATCGAACAGTGGGTTCCACC
AGCTCCTAGACCTGCTCAGAGAAATAGAAGGGTGAACCTGCTTGTGAGCCACCTTTGTT
CTCCAGGTGATATCGAGTGGCTTAGGCGTTGCGTTGAGGCTTTTGGACTTCAGCCTATCA
TCCTGCCTGATCTGGCTCAGTCTATGGATGGTCATCTTGCTCAGGGCGATTTCAGCCCTC
TTACTCAAGGTGGTACTCCTCTTAGGCAGATTGAGCAGATGGGACAGTCTCTGTGCTCTT
TCGCTATTGGTGTGTCTCTGCACAGGGCTTCATCTTTGCTTGCTCCTAGATGCAGAGGTG
AGGTGATCGCTTTGCCTCACCTTATGACTCTTGAGAGGTGCGACGCTTTCATTCACCAGC
TGGCTAAGATTTCTGGTAGGGCTGTTCCTGAGTGGCTTGAGAGACAAAGGGGTCAACTT
CAGGACGCTATGATCGACTGCCATATGTGGCTTCAGGGTCAGAGAATGGCTATTGCTGC
TGAGGGTGATCTTCTTGCTGCTTGGTGCGATTTCGCTAACAGTCAAGGTATGCAGCCTGG
TCCTCTTGTTGCTCCTACTGGTCATCCTTCACTTAGGCAGTTGCCTGTTGAAAGGGTTGTG
CCTGGTGATCTTGAGGATCTTCAGACCCTTCTTTGCGCTCACCCTGCTGATCTTTTGGTGG
CTAATTCTCACGCTAGGGACCTTGCTGAGCAGTTTGCTTTGCCTTTGGTGAGAGCTGGAT
TCCCGCTTTTCGATAAGCTTGGCGAGTTCAGAAGAGTGAGGCAGGGATATTCTGGCATG
AGGGATACCCTTTTCGAGCTGGCAAACCTGATTAGAGAGAGGCATCATCACCTGGCTCA
CTACAGATCTCCATTGAGGCAGAACCCTGAGTCCTCTCTTTCTACTGGTGGTGCTTATGC
TGCTGAT

>nifQ_SN141_SN209

ATGCCTCCTCTTGATTGGCTTCGTAGGCTTTGGCTTCTTTACCACGCTGGTAAGGGCTCAT
TCCCTCTTAGGATGGGACTGTCTCCTAGAGATTGGCAGGCTCTTAGAAGAAGGCTTGGC
GAAGTTGAGACTCCTCTGGATGGTGAGACTCTTACTAGGCGGAGACTTATGGCTGAGCT
TAACGCTACTAGAGAGGAAGAAAGGCAGCAGCTTGGTGCTTGGCTTGCTGGTTGGATGC
AACAAGATGCTGGTCCTATGGCTCAGATTATCGCTGAGGTGAGCCTTGCTTTCAACCACC
TTTGGCAGGATCTTGGTCTTGCTTCTAGGGCTGAGCTGAGGCTTCTTATGTCTGATTGCTT
CCCTCAGCTGGTGGTGATGAACGAGCATAACATGCGGTGGAAGAAGTTCTTCTACAGGC
AGAGATGCCTTCTGCAGCAGGGTGAAGTTATTTGCCGGTCACCTTCTTGTGATGAGTGCT
GGGAAAGAAGCGCTTGCTTCGAA

>nifS_SN31_SN210

ATGAAGCAGGTCTACCTGGACAACAATGCTACCACCAGGCTTGATCCTATGGTGCTCGA
AGCTATGATGCCTTTCCTGACCGATTTCTACGGCAACCCCTCTAGCATTCACGATTTCGG
TATTCCAGCTCAGGCTGCTCTTGAAAGGGCTCATCAACAAGCTGCTGCTTTGCTTGGTGC
TGAGTACCCGTCTGAGATCATCTTCACATCTTGCGCTACTGAGGCTACCGCTACTGCTAT
TGCTTCTGCTATCGCTCTGCTTCCTGAGCGGAGAGAGATTATCACCTCTGTTGTTGAGCA
CCCTGCTACCTTGGCTGCTTGTGAACATCTTGAGAGGCAGGGCTACAGAATCCACAGGA
TTGCTGTTGATTCTGAGGGCGCTCTTGACATGGCTCAGTTTAGGGCTGCTTTGTCTCCTA
GAGTGGCTCTGGTTTCTGTGATGTGGGCTAACAATGAGACTGGCGTGCTGTTCCCTATTG
GTGAGATGGCTGAACTTGCACACGAACAGGGTGCACTTTTCCACTGTGATGCTGTTCAG
GTGGTGGGCAAGATTCCTATTGCAGTTGGTCAGACCAGGATCGACATGCTGTCTTGCTCT
GCTCATAAGTTCCACGGTCCTAAAGGTGTGGGTTGCCTTTACCTTAGAAGGGGAACTAG
ATTCAGGCCTCTGCTTAGAGGTGGTCACCAAGAGTATGGTAGACGTGCTGGAACCGAGA
ACATCTGCGGTATCGTTGGTATGGGTGCTGCTTGCGAGCTTGCTAACATTCACTTGCCTG
GTATGACCCACATTGGCCAGCTTAGGAATAGGCTTGAGCACAGGCTTTTGGCCTCTGTGC
CTTCTGTTATGGTTATGGGTGGTGGTCAGCCTAGAGTTCCTGGTACTGTGAATCTGGCCT
TCGAGTTCATTGAAGGCGAGGCTATTCTGCTGCTTCTGAACCAGGCTGGTATCGCTGCTT
CTTCTGGTTCTGCTTGCACCTCTGGTTCTTTGGAGCCTTCTCATGTGATGCGGGCAATGA
ACATTCCTTACACTGCTGCTCACGGGACCATCAGGTTTTCTTTGTCTAGGTACACCCGTG
AGAAAGAAATCGACTACGTGGTGGCTACCCTGCCTCCTATTATTGATAGGCTTAGGGCT
CTGTCCCCGTACTGGCAAAATGGTAAGCCTAGACCTGCAGACGCTGTGTTCACTCCTGTT
TATGGT

>nifU_SN32_SN211

ATGTGGAACTACAGCGAGAAGGTGAAGGACCACTTCTTCAACCCTAGGAACGCTAGAGT
GGTGGACAATGCTAATGCTGTGGGTGATGTGGGTTCTCTGTCTTGCGGTGATGCTCTTAG
GCTTATGCTTAGGGTTGACCCGCAGTCTGAGATCATTGAAGAGGCTGGTTTCCAGACCTT
CGGTTGCGGTTCTGCTATCGCTTCTTCTAGCGCTCTTACCGAGCTTATCATTGGTCACACT
CTTGCTGAGGCTGGCCAGATTACTAACCAGCAGATTGCTGATTACCTGGACGGTTTGCCT
CCTGAGAAGATGCATTGCTCTGTGATGGGTCAAGAGGCTCTTAGGGCTGCAATTGCTAA
TTTCAGGGGCGAGTCTCTGGAAGAGGAACACGATGAAGGTAAGCTGATCTGCAAGTGCT
TCGGTGTGGATGAGGGTCACATTAGAAGGGCTGTGCAGAACAACGGTCTTACCACTTTG
GCTGAGGTGATCAACTACACCAAGGCTGGTGGTGGTTGTACTTCTTGCCACGAGAAGAT
TGAGCTTGCTCTGGCTGAGATTCTTGCTCAGCAACCTCAAACTACTCCTGCTGTGGCTTC
TGGTAAGGATCCTCATTGGCAGTCTGTCGTGGACACCATTGCTGAACTTAGGCCTCACAT
TCAGGCTGATGGTGGCGATATGGCTCTTTTGTCTGTGACCAACCATCAGGTGACCGTGTC
TTTGTCTGGTTCATGCTCTGGTTGCATGATGACCGATATGACCCTTGCTTGGCTGCAGCA
AAAGCTGATGGAAAGGACTGGCTGCTACATGGAAGTTGTGGCTGCT

>nifV_SN142_SN212

ATGGAACGGGTGCTGATCAACGATACCACCTTGAGAGATGGTGAGCAGTCTCCTGGTGT
TGCTTTCAGGACTTCTGAGAAGGTGGCAATTGCTGAGGCTCTTTACGCTGCTGGTATCAC
CGCTATGGAAGTTGGTACTCCTGCTATGGGTGACGAAGAGATTGCTAGGATTCAGCTGG
TGAGAAGGCAGCTTCCTGATGCTACTCTTATGACCTGGTGCAGGATGAACGCTCTTGAG
ATTAGGCAGTCTGCTGATCTGGGCATCGATTGGGTTGACATCTCTATCCCTGCTAGCGAT
AAGCTGAGGCAGTACAAGCTTAGAGAGCCTCTTGCTGTTCTGCTTGAGAGGCTGGCTAT
GTTCATTCACCTTGCTCACACCCTGGGTCTGAAGGTGTGCATTGGTTGTGAGGATGCTAG

CAGAGCTTCTGGTCAGACCCTTAGGGCTATTGCTGAAGTGGCTCAAAATGCTCCTGCTGC
TAGGCTTAGGTACGCTGATACTGTGGGTCTGCTTGATCCTTTCACTACCGCTGCTCAGAT
TAGCGCTCTTAGGGATGTTTGGTCTGGCGAGATTGAGATGCACGCTCACAACGATCTTG
GTATGGCTACCGCTAATACCCTGGCTGCTGTTTCTGCTGGTGCTACTTCTGTGAATACCA
CCGTGCTTGGTCTTGGTGAGAGAGCTGGTAACGCTGCTGCTTGGAAGCCTTCTGCTCTTG
GTTTGGAGAGATGCCTGGGTGTTGAGACTGGTGTTCACTTCTCTGCTTTGCCTGCTCTTTG
CCAGAGAGTTGCTGAAGCTGCACAGAGGGCAATTGATCCTCAACAGCCTCTTGTTGGCG
AGCTTGTGTTCACACATGAGTCTGGTGTGCATGTGGCTGCTCTGCTTAGAGACTCTGAGA
GCTACCAGTCTATCGCCCCATCTCTTATGGGCAGATCTTACAGGCTTGTGCTGGGTAAGC
ACTCTGGTAGGCAAGCTGTTAACGGTGTGTTCGATCAGATGGGCTACCACCTTAATGCC
GCTCAGATCAACCAGCTTCTGCCTGCTATTAGACGGTTCGCTGAGAACTGGAAGAGGTC
CCCTAAGGATTACGAGCTGGTGGCTATCTACGATGAGCTTTGTGGTGAGTCCGCTCTTAG
AGCTAGAGGT

>nifW_SN143_SN213

ATGATGGAATGGTTCTACCAGATTCCTGGTGTGGACGAGCTTAGGTCTGCTGAGTCATTC
TTCCAGTTCTTCGCTGTGCCTTACCAGCCTGAGCTTCTTGGTAGATGCTCTCTTCCTGTGC
TTGCCACCTTCCATAGAAAGCTTAGGGCTGAAGTGCCTCTGCAGAACAGGCTTGAGGAT
AATGATAGGGCTCCTTGGCTTCTGGCTAGAAGGCTTCTTGCTGAGTCTTATCAGCAGCAG
TTCCAAGAGTCTGGAACT

>nifX_SN144_SN214

ATGCCTCCTATCAACAGGCAGTTCGACATGGTGCATTCCGACGAGTGGTCTATGAAGGT
GGCATTCGCTAGCAGCGATTACAGGCATGTGGATCAGCATTTCGGTGCTACTCCTAGGCT
TGTTGTGTACGGTGTGAAGGCTGATAGGGTGACCCTTATTAGAGTGGTGGACTTCAGCG
TTGAGAACGGTCATCAGACCGAGAAGATCGCTAGAAGGATTCACGCTCTCGAGGATTGC
GTGACCCTTTTCTGTGTGGCTATTGGGGATGCTGTGTTCAGGCAGCTTCTTCAGGTTGGA
GTTAGGGCTGAAAGAGTGCCTGCCGATACAACCATTGTGGGTCTGCTTCAAGAGATCCA
GCTGTACTGGTACGATAAGGGCCAGAGAAAGAATCAGAGGCAGAGAGATCCTGAGAGG
TTCACCAGATTGCTGCAAGAGCAAGAGTGGCACGGTGATCCTGATCCTAGAAGA

>nifY_SN145_SN215

ATGAGCGATAACGACACCCTGTTTTGGAGGATGCTTGCCCTTTTCCAGTCTCTGCCTGAT
CTTCAACCTGCTCAGATTGTGGATTGGCTGGCTCAAGAATCTGGTGAGACTCTTACTCCT
GAGAGGCTTGCTACTCTTACCCAACCTCAACTGGCTGCTTCTTTCCCTTCTGCTACTGCTG
TTATGTCTCCTGCTAGGTGGTCTAGAGTGATGGCTTCTCTTCAGGGTGCACTTCCTGCTC
ACCTTAGAATTGTGAGGCCAGCTCAAAGGACCCCTCAACTTCTTGCTGCTTTCTGCTCTC
AGGATGGCCTTGTGATTAACGGTCATTTCGGTCAGGGCCGTCTGTTCTTCATCTACGCTT
TTGATGAACAAGGCGGCTGGCTGTACGATCTTAGGCGTTATCCTTCTGCTCCTCACCAGC
AAGAGGCTAATGAAGTGAGAGCTAGGCTGATCGAGGATTGCCAGCTTTTGTTCTGCCAA
GAGATTGGTGGTCCTGCTGCTGCTAGGCTTATTAGGCATAGGATTCACCCTATGAAGGCT
CAGCCTGGAACTACCATTCAGGCTCAGTGTGAGGCTATTAACACCCTTTTGGCTGGTAGG
TTGCCTCCATGGCTTGCTAAGAGGCTTAACAGGGATAACCCGCTGGAAGAGAGGGTTTT
C

>nifZ_SN146_SN216

ATGAGGCCGAAGTTCACCTTCTCTGAAGAGGTTAGAGTGGTGCGGGCTATTAGGAACGA
TGGTACTGTTGCTGGTTTCGCTCCTGGTGCTCTTTTGGTTAGAAGGGGTTCTACCGGCTTC
GTGAGAGATTGGGGAGTTTTCTTGCAGGACCAGATTATCTACCAGATTCACTTCCCCGAG
ACTGACAGGATTATCGGCTGTAGAGAGCAAGAGCTGATCCCTATTACTCAGCCTTGGCT
TGCTGGTAACCTGCAGTACAGGGATTCTGTGACTTGCCAGATGGCTCTTGCTGTGAATGG
TGATGTTGTGGTGTCTGCTGGTCAGAGGGGTAGAGTTGAAGCTACTGATAGAGGTGAGC
TGGGCGATTCTTACACCGTGGATTTTAGCGGCAGATGGTTCAGGGTTCCAGTGCAGGCT
ATTGCTCTTATCGAAGAGAGGGAAGAG
